# Ecological niche and phylogeography elucidate complex biogeographic patterns in *Loxosceles rufescens* (Araneae, Sicariidae) in the Mediterranean Basin

**DOI:** 10.1186/s12862-014-0195-y

**Published:** 2014-10-09

**Authors:** Enric Planas, Erin E Saupe, Matheus S Lima-Ribeiro, A Townsend Peterson, Carles Ribera

**Affiliations:** Institut de Recerca de la Biodiversitat (IRBio), Departament de Biologia Animal, Universitat de Barcelona, Barcelona, Spain; Department of Geology, University of Kansas, Lawrence, Kansas USA; Biodiversity Institute, University of Kansas, Lawrence, Kansas USA; Departamento de Ciências Biológicas, Universidade Federal de Goiás, Campus Jataí, Jataí, GO Brasil

**Keywords:** Arachnid, Evolution, Human-mediated dispersal, Pleistocene glaciations, Refugia, Spider

## Abstract

**Background:**

Understanding the evolutionary history of morphologically cryptic species complexes is difficult, and made even more challenging when geographic distributions have been modified by human-mediated dispersal. This situation is common in the Mediterranean Basin where, aside from the environmental heterogeneity of the region, protracted human presence has obscured the biogeographic processes that shaped current diversity. *Loxosceles rufescens* (Araneae, Sicariidae) is an ideal example: native to the Mediterranean, the species has dispersed worldwide via cohabitation with humans. A previous study revealed considerable molecular diversity, suggesting cryptic species, but relationships among lineages did not correspond to geographic location.

**Results:**

Delimitation analyses on cytochrome *c* oxidase subunit I identified 11 different evolutionary lineages, presenting two contrasting phylogeographic patterns: (1) lineages with well-structured populations in Morocco and Iberia, and (2) lineages lacking geographic structure across the Mediterranean Basin. Dating analyses placed main diversification events in the Pleistocene, and multiple Pleistocene refugia, identified using ecological niche modeling (ENM), are compatible with allopatric differentiation of lineages. Human-mediated transportation appears to have complicated the current biogeography of this medically important and synanthropic spider.

**Conclusions:**

We integrated ecological niche models with phylogeographic analyses to elucidate the evolutionary history of *L. rufescens* in the Mediterranean Basin, with emphasis on the origins of mtDNA diversity. We found support for the hypothesis that northern Africa was the center of origin for *L. rufescens*, and that current genetic diversity originated in allopatry, likely promoted by successive glaciations during the Pleistocene. We corroborated the scenario of multiple refugia within the Mediterranean, principally in northern Africa, combining results from eight atmosphere–ocean general circulation models (AOGCMs) with two different refugium-delimitation methodologies. ENM results were useful for providing general views of putative refugia, with fine-scale details depending on the level of stringency applied for agreement among models.

**Electronic supplementary material:**

The online version of this article (doi:10.1186/s12862-014-0195-y) contains supplementary material, which is available to authorized users.

## Background

The Mediterranean Basin was placed among 25 world biodiversity ‘hotspots for conservation priority’ based on high levels of endemism and rapid loss of natural areas [1]. Humans began transforming Mediterranean ecosystems >10,000 years ago [2], such that today, only 4.7% of primary vegetation remains unaltered in the region [3]. Despite these long-standing impacts, the Mediterranean continues to be home to a diverse flora and fauna.

Several factors (e.g. climate, geology) promoted development of this diversity at different temporal and geographic scales, such as the Messinian Salinity Crisis and the onset of a Mediterranean-type climate ~3.2 Ma [4]. Glaciations during the Pleistocene (~2.6 – 0.02 Ma) also played a role in shaping current diversity patterns [5]: climatic fluctuations during this period caused regional extinctions [6,7] and promoted range shifts and diversification via allopatric speciation (e.g. [8]). The three major southern Mediterranean peninsulas (Iberia, Italy, Balkans) were long thought to have served as major refugia for European flora and fauna during Pleistocene glaciations [8], but recent studies have challenged this paradigm as too simplistic to explain observed patterns [9,10]. As a consequence, some authors have argued for refugia within refugia [11] or multiple northern refugia ([12,13]; but see [14]).

Different approaches have been used to delimit glacial refugia in the Mediterranean. Traditionally, paleoecological evidence [15-17] and concentrations of endemic taxa [18] served as primary evidence, but more recently, comparative phylogeographic studies have been used to delineate “phylogeographic hotspots”, or areas with unique genetic diversity [5], while others have used ecological niche models (ENMs) in conjunction with paleoclimate simulations (e.g. [19]). The latter methodology projects environmental requirements of species onto past conditions, thus offering an approach that is independent and complementary [20-22]. Each approach has drawbacks and merits, but identifying regions using multiple approaches offers increased confidence [21,23,24]. Although other studies have successfully integrated phylogeographic and ENM approaches to uncover putative refugial areas [25-27], few have treated the entire Mediterranean Basin [19,28,29], and none have considered the added complexity of a human commensal.

Two *Loxosceles* spider species coexist in the Mediterranean: *L. rufescens* (Dufour 1820) and the Tunisian *L. mrazig* Ribera and Planas 2009. *Loxosceles rufescens* originated in the Mediterranean [30-34] but has been transported worldwide by humans [30-32,34,35]. Duncan et al. [32] documented diverse genetic lineages among individuals morphologically consistent with *L. rufescens*, suggesting cryptic speciation. The morphological simplicity within *Loxosceles* makes traditional species delimitation “singularly difficult” ([36] p. 142), so genetically-based methodologies are key to illuminating the evolutionary history of this group. In addition, relationships between *L. rufescens* lineages are not predictable by geographic location [32], which contrasts with the high spatial structure of populations in other *Loxosceles* species [31,34]. Therefore, *Loxosceles rufescens* is an ideal model to unravel the role of climatic changes and human impacts on the evolutionary history of Mediterranean species.

In this contribution, we examine mtDNA diversity within *L. rufescens*, and elucidate evolutionary processes that promoted this diversity via a combination of phylogeographic and ENM approaches. Our working hypothesis is that current mtDNA diversity was generated allopatrically in glacial refugia across the Mediterranean, and that *L. rufescens* biogeography was since obfuscated by human activity. This hypothesis offers three opportunities for testing: (1) divergence times should coincide with periods of repeated glaciations (~2.6 – 0.02 My), (2) multiple putative refugia should have existed to provide areas of origin for distinct evolutionary lineages, and (3) widespread lineages should show no spatial structure within the Mediterranean Basin.

## Methods

### Taxonomic sampling

**Figure 1:**
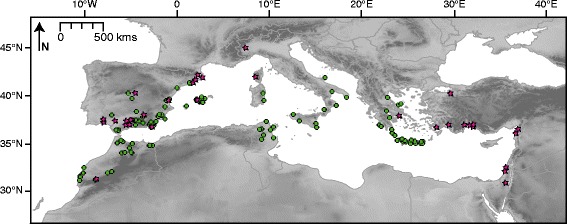
**Map of sampling localities.** Green circles represent localities used in ENM analyses; pink stars indicate additional sampled localities used in the phylogenetic analyses.

We sampled *L. rufescens* populations across the Mediterranean Basin to replicate and complement previous sampling [32] and to increase the likelihood of discovering new lineages. In all, 158 localities were sampled across eight countries (Figure 1 and Additional file 1). From these localities, 310 individuals were sequenced and included in our analyses.

### Molecular data

We included at least one individual from each locality in molecular analyses. Total genomic DNA was extracted using SPEEDTOOLS Tissue DNA Extraction Kit (BIOTOOLS) following manufacturer’s protocols. We amplified a portion of the cytochrome *c* oxidase subunit I (*cox1*) using LINF and GAYAR primers [34], producing 1016-bp fragments; we used different combinations of C1-J-1718, C1-J-2183 [37] and C1-N-2191 [38] internal primers when the first set failed. PCR reactions were conducted at a final volume of 25 μL using either *Taq* polymerase (Promega) or Biotools *Pfu* DNA Polymerase (Biotools). PCR products were cycle-sequenced in both directions using the same PCR primers and the BigDye Terminator v.3.1 Cycle Sequencing Kit (Applied Biosystems). Sequences were derived from these products in an ABI 3700 automated sequencer at the Serveis Científico-Tècnics of the Universitat de Barcelona and in Macrogen, Inc. (Seoul, Korea). Raw sequences were edited and assembled with GENEIOUS v.4.6.5 [39]. To avoid amplification of pseudogenes reported by Duncan et al. [32] for *Loxosceles cox1*, we mostly used *Loxosceles*-specific primers; we also translated sequences into amino-acids, and checked for stop-codons.

Sequences were aligned unambiguously in GENEIOUS using ClustalW [40] with default parameters. We partitioned data by codon position and explored best partitioning schemes and substitution models simultaneously using PartitionFinder v.1.0.1 [41] under a Bayesian information criterion for the entire matrix. These steps were conducted independently for individuals employed in delimitation analyses, and for the reduced set used in dating analyses.

### Phylogenetic analyses

We used maximum likelihood (ML) and Bayesian inference (BI) to infer phylogenetic relationships from a dataset containing one representative of each *cox1* haplotype. ML analyses were conducted in RAxML v.7.4.2 [42] with the aid of the graphical front-end RAXML-GUI v.1.3 [43]. We applied a rapid hill-climbing search algorithm, and conducted 1000 non-parametric bootstrap replicates. BI analyses were conducted in MrBayes v.3.2 [44] with two independent runs of two million generations with four Markov chains (one cold, three heated), sampling every 1000 generations. We checked convergence of chains visually in Tracer v.1.6 [45] until effective sample sizes (EES) were above 200, and the average standard deviations of split frequencies (ASDSF) of the two runs were below 0.01. The first 25% of trees in each run were discarded as burn-in, and a majority-rule consensus tree was generated from remaining trees. BI trees were also obtained with BEAST v.1.7.4 [46] using a coalescent tree prior with a constant population size and a relaxed lognormal clock (rate fixed arbitrarily at one). Two independent runs of 20 million generations (sampling every 1000th generation) were used for each analysis. We assessed convergence and correct mixing of chains by inspecting the trace plots and ensuring EES > 200 in Tracer. The two runs were combined using LogCombiner and TreeAnotator [47] after removing a 10% burn-in of the samples. Position of the root of the tree was estimated implicitly in BEAST [48] and used for rooting RAxML and MrBayes trees.

Genetic p-distances between and within lineages (see Molecular delimitation analyses) were calculated using MEGA5 [49]. To study demographic history (only for lineages with *N* > 10), we applied two neutrality tests: Fu’s *F*_S_ [50] and *R*_2_ [51] in DnaSP v.5.10 [52]. We assessed statistical significance and confidence intervals using coalescence simulations in DnaSP with 1000 replicates and default parameters. We excluded six sequences from the A6 lineage that lacked the data for the 3' or 5' ends.

### Molecular delimitation analyses

Morphological traits provide few means of distinguishing lineages of *L. rufescens*, but genetic distances between lineages are high and different lineages are often sympatric at micro-scales ([32]; pers. obs.); consequently, assignment of individuals to lineages is not a simple function of geographic location. To account for this, we used two methodologies for objective delimitation of evolutionary lineages: (1) a General Mixed Yule Coalescent model (GMYC), and (2) phylogenetic network estimation using statistical parsimony (TCS). Although these methodologies have been successful at circumscribing species and often yield results congruent with alternative species delimitation methods (e.g. based on morphology, behavior; [53,54]), their utility depends on different factors (e.g. effective population size, sampling scheme; [53,55,56]), with results tending perhaps towards overestimation of species numbers [57].

#### GMYC

GMYC is a species delimitation method that provides an objective way to delimit genetic clusters [56]. The method was developed to identify putative species in poorly-known groups based on single molecular markers [58]. The model seeks transition points (thresholds) between inter-specific relationships and intra-specific coalescent events, and subsequently tests the likelihood of the model against a null model that assumes a single branching process for the entire tree [58]. Since GMYC requires identical sequences to be removed [56], we included one representative of each haplotype. Because GMYC is sensitive to relative branch lengths and topology of the ultrametric tree [54], we explored effects of alternative input trees obtained from ML using RAxML, and BI using MrBayes and BEAST, as described below.

We generated a ML tree using RAxML [42], as outlined above. We converted this result to an ultrametric tree using PATHd8 [59], arbitrarily fixing the root at 100 units. Bayesian trees were derived using MrBayes [44], with two independent runs of 50 million generations with four Markov chains each (one cold, three heated), sampling every 1000 generations with a coalescent clock prior. The clock rate was arbitrarily fixed to one, and we used a lognormal distribution as a prior for population size. We checked for chain convergence as described above. The ultrametric tree was imported into R [60] and made fully dichotomous with the multi2di function in the APE package v.30.8 [61]. The ultrametric Bayesian tree from BEAST was obtained as outlined above.

#### TCS

Separate haplotype networks in statistical parsimony analyses might provide a useful and objective method to delimit individuals into evolutionary significant units [62]. Networks delimited using a 95% parsimony connection limit, i.e. the probability that two DNA sequences share a parsimonious relationship without multiple substitutions underlying any single nucleotide difference [63], generally correspond to single species (78% in 663 examples in [62]). We obtained statistical parsimony networks in TCS v.1.3 [64] using our complete dataset (310 individuals), applying 95% and 99% connection limits, and treating gaps as missing data.

### Patterns of genetic diversity

We investigated how mtDNA diversity is distributed across geography within *L. rufescens* following [65]; this methodology is of particular use when localities have unequal sample sizes. Diversity statistics are computed by considering samples located within a perimeter around a grid point. We set grid points every 100 km in latitude and longitude, and computations were assessed across random sets of five individuals, bootstrapping 1000 times. We calculated three diversity indices, total diversity (H_T_), haplotype richness (H_R_), and rarity index (R) [19], in R [60] using custom scripts provided by N. Arrigo. High genetic diversity combined with rare haplotypes are characteristics of populations with a long *in situ* history, so identification of this pattern may indicate regions of origin for the different lineages [66]. We explored effects of different parameters on diversity indices, varying grid point distances 50–450 km, and numbers of individuals per analysis 3–10.

We analyzed the geographic structure of each lineage using a Mantel test implemented in the Isolation By Distance Web Service v.3.23 [67]. Geographic distances among localities were calculated using the Geographic Distance Matrix Generator v.1.2.3 [68], and genetic p-distances between localities were calculated in MEGA. We performed Mantel tests with 999 permutations to assess significance of correlations between genetic distances and log-transformed geographic distances. We excluded lineages B1 and B3 from Mantel tests owing to low numbers of localities (< five). Lineages A1 to A5 were pooled for the analyses (see results), and remaining lineages were assessed independently.

### Dating analysis

Since we lacked reliable calibration points within the *L. rufescens* lineage, we explored two divergent rates. First, we used a *Loxosceles*-specific molecular rate obtained using fossil and island ages as calibration points [34]. Second, we applied the substitution rate obtained for the same mtDNA gene in a closely-related spider family (Dysderidae; [69]). As the two rates are fairly divergent, we suspect that actual divergence times for *L. rufescens* fall somewhere between these end points. Rates were incorporated as priors under a normal distribution with mean 0.095 ± 0.001 and 0.0199 ± 0.001, respectively. Dating analyses were conducted in BEAST [46], using an uncorrelated lognormal relaxed clock [48] and a Yule tree prior. One representative of each lineage was included in analyses, and we used two independent runs of 10 million generations, sampling every 1000th generation, for each analysis. We assessed convergence and correct mixing of chains by inspecting trace plots and ensuring EES > 200 in Tracer. Runs were combined using LogCombiner and TreeAnotator, after removing a 10% burn-in.

### Ecological Niche Modeling (ENM)

#### Study area

Niche models for *L. rufescens* were calibrated within a region that we hypothesized was ‘sampled’ by the species over its relevant history; in other words, a region the species had been able to deem suitable/unsuitable (**M**; *sensu* Barve et al. [70]), intersected with regions that were sampled as part of this study [71]. To calculate **M**, we buffered *L. rufescens* records by the longest distance from the sea to a documented locality (~350 km), which provided an estimate of the dispersal capability of the species. We excluded areas that we were unable to sample, or where closely-related species occur (*L. mrazig* in the southern parts of Tunisia and southeastern Morocco; an undescribed species group in the Sous Valley of Morocco). These steps left a calibration area comprising Morocco, the Iberian Peninsula, Balearic Islands, Sardinia, Sicily, continental Italy, Greece and adjacent islands, Crete, and Tunisia. After model calibration in this area, we projected results to the entirety of the Mediterranean Basin, within a bounding rectangle of 48.2–26.7° latitude and −14.8–41.2° longitude (see Figure 1).

#### Climate data

We obtained climate data from eight coupled atmosphere–ocean general circulation model (AOGCMs) simulations: Community Climate System Model (CCSM), Centre National de Recherches Météorologiques (CNRM), Consortium for Small-scale Modelling (COSMOS), Goddard Institute for Space Studies (GISS), Institute Pierre Simon Laplace (IPSL), Model for Interdisciplinary Research on Climate (MIROC), Max-Planck Institut für Meterologie (MPI), and Meteorological Research Institute (MRI). These AOGCMs were derived from the multi-model ensemble in the Coupled Model Intercomparison Project Phase 5 (CMIP5) [72] and the Paleoclimate Modelling Intercomparison Project Phase 3 (PMIP3) [73]; more details about the climate models are provided in Additional file 2 and in Taylor et al. [74].

We downloaded monthly simulation outputs for annual precipitation and mean, maximum, and minimum temperatures from the pre-industrial experiment, which characterized current climatic conditions. Past conditions were characterized using paleoclimate simulations for the mid-Holocene (~6 Ka) and Last Glacial Maximum (LGM, ~21 Ka) from each AOGCM, with the exception of GISS and COSMOS, which lacked mid-Holocene outputs. To produce climate scenarios at resolutions relevant to the spatial scale of species’ distributions, we downscaled climate layers to 0.5° resolution using a standard change-factor approach [75]: (1) for each AOGCM, we computed the difference between the past (mid-Holocene and LGM) and current simulations (i.e. pre-industrial), and this difference (i.e. climate change trends) and the current climate were interpolated to 0.5° spatial resolution using kriging; (2) these differences were added to the interpolated current climate to obtain interpolated past conditions. We used absolute differences for temperatures and relative differences for precipitation; see [76] for further details. This procedure maintains higher-resolution topography in downscaled climates and ensures coherency of climatic patterns over time [77]. From these downscaled climatic scenarios, we computed 19 so-called ‘bioclimate’ variables [71], but we excluded mean temperature of the wettest and driest quarters, and precipitation of the warmest and coldest quarters, owing to the spatial artifacts that emerge in these four variables.

For each AOGCM, we performed a principal components analysis in R [60] on the 15 bioclimatic variables in the calibration area to create new axes that summarized variation in fewer, independent dimensions, and to reduce co-linearity among variables. We retained those principal components that explained cumulatively 99% of the overall variance in the dataset (i.e. the first six principal components for all AOGCMs except GISS, which required only the first five) for model calibration. These principal components were used to calculate corresponding composite variables for mid-Holocene and LGM conditions. The PCA structure for current conditions was enforced for the past conditions using a script in R [60] written by A. Lira and N. Barve (U. Kansas).

#### Occurrence data

We used a subset (130 records) of occurrence data associated with samples employed in the genetic analyses. These localities were obtained directly from fieldwork in natural areas by EP and others (see Acknowledgments), and have precise latitude/longitude coordinates derived from GPS measurements. To consider the potential biasing effects introduced by spatial autocorrelation, such that spatially-clumped points would over-represent certain environments, we calculated spatial lags in environmental data using Geostatistical Analyst in ArcMap v.10 (ESRI, Redlands, CA), and subsampled the records to create 10 replicate datasets using a script in R [60] written by N. Barve (U. Kansas). Based on lag calculations, we enforced a minimum distance of 50 km between localities. Subsampling occurrence data to account for environmental lag ensures that suitable conditions are evenly weighted during model calibration. Each subset included 62–65 occurrence records.

#### Modeling algorithm

ENMs were generated using Maxent v.3.1.1 [78], which can be monitored for extrapolation errors when projecting to past climates [79,80]. Maxent minimizes the relative entropy between two probability densities—one from the distributional data and one from the background or study area—defined in covariate space [81]. We used default parameters, but specified 100 bootstrap replicates per occurrence dataset and a minimum training presence threshold rule to avoid omission error. We took the median of the 100 runs per occurrence dataset multiplied by 1000, and converted to integer grids in ArcMap. These grids were used to calculate the median of the 10 subsets for each AOGCM. The resulting models were converted to binary grids based on all 130 localities using a minimum training present approach [82]. Use of multiple AOGCMs [83] provides a broader estimate of suitable conditions for *L. rufescens*, but we acknowledge that ensemble-modeling approaches may shed additional light on model-dependent results (see [24]).

When transferring models temporally or spatially, conditions outside the range of climatic values in the calibration region (**M**) may be encountered, leading to situations of extrapolation. To identify these regions, we used a script in R [60] written by N. Barve [84] to create Mobility Oriented Parity (MOP) maps [80]. Areas identified as both suitable and extrapolative were removed from analyses to avoid interpreting results outside of known climatic response conditions for *L. rufescens*.

#### Model evaluation

To evaluate the predictive power of the models, we partitioned two of the replicate occurrence datasets at random: half of the data was used in model calibration, and the other half for model testing via partial Receiver Operator Characteristic (partial ROC) approaches [82]. Partial ROC avoids many of the problems associated with traditional ROC analyses, such as equal weighting of omission and commission errors, and consideration of model thresholds that yield irrelevant predictions. These tests were run using a Visual Basic routine developed by N. Barve [85], with an expected error rate of *E* = 1% [82]. We performed 1000 bootstrap iterations by resampling 50% of test points with replacement.

### Identifying refugial areas

We identified possible refugia as areas that remained continuously suitable from the LGM to the present. Because glaciations were common throughout the Pleistocene, with glacial and interglacial conditions recurring in nearly-regular cycles with similar amplitudes (at least since 800 Ka) [86], we assume that the three time slices used here (LGM, mid-Holocene and present) capture, at least to some degree, the key climatic conditions across the entire Pleistocene [87,88]. We acknowledge, however, that these reconstructions are merely broad estimates of potential refugial conditions for *L. rufescens* and lack constraining data for the earlier half of the epoch.

**Figure 2:**
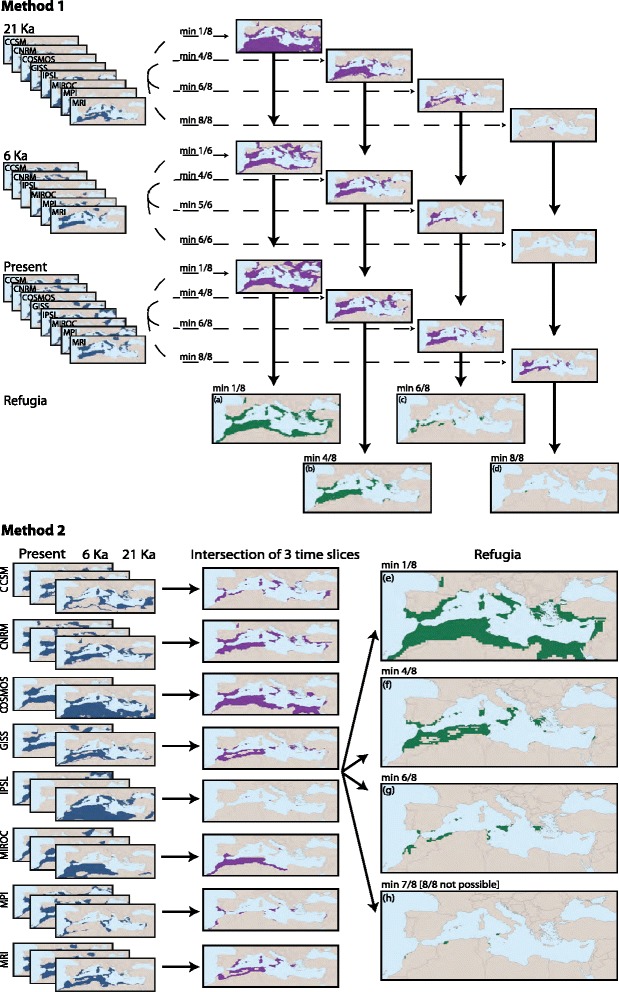
**Refugium delimitation methods.** Refugial areas were identified as the intersection of the three time slices. Method 1 sought consensus among the eight AOGCMs in each time slice by requiring at least **(a)** one to agree, **(b)** 4/8 to agree, **(c)** 6/8 to agree and **(d)** all 8 to agree. Method 2 sought the intersection of the three time slices for each AOGCM independently, subsequently requiring the AOGCMs to agree in the same fashion as M1: at least **(e)** one to agree, **(f)** 4/8 to agree, **(g)** 6/8 to agree and **(h)** 7 to agree (8/8 was not possible).

We identified refugia using two approaches: approach one (M1) required AOGCMs to agree on suitable area for each time slice, applying four different thresholds (8 of 8 to AOGCMs agree, 6/8, 4/8, and 1/8). This resulted in four different suitability maps for the LGM, the mid-Holocene, and the present-day. The intersection of the three time slices was taken as the final refugial area, which created four different possible refugial scenarios (Figure 2a-d). The second approach (M2) calculated refugial area across the three time slices for each individual AOGCM (Figure 2e-h); in other words, the intersection of suitable area was taken across the three time slices (LGM, mid-Holocene, and present-day) for each AOGCM independently. From the individual AOGCM maps of refugial areas, we applied the threshold criteria of M1 to identify consensus regions (8 of 8 AOGCMs agree, 6/8, 4/8 and 1/8). In effect, these two methods explored sensitivity to threshold choice, and resulted in eight putative refugial maps. We repeated the two methods without COSMOS and IPSL, as these AOGCMs often exhibit anomalous climatic patterns compared to other AOGCMs. Using ENM to identify putative refugia can elucidate potential divergence mechanisms. Considerable caution, however, should be exercised in interpreting such analyses, particularly in light of the spatial grain of these data: coarse-resolution climate data cannot detect fine-scale phenomena (i.e. microrefugia *sensu* Rull [89]).

## Results

### Phylogenetic analyses

New sequences obtained during this study were deposited in GenBank with accession numbers KJ560560 - KJ560863 (Additional file 1); additional sequences were downloaded from GenBank (Additional file 1). In total, 310 sequences were used, containing 63 different haplotypes. PartitionFinder suggested a non-partitioned codon scheme with a HKY + G substitution model as the best fit for these data under the Bayesian information criterion; we used this partition scheme and substitution model in all phylogenetic analyses except RAxML, where only a GTR + G model was available.

**Figure 3:**
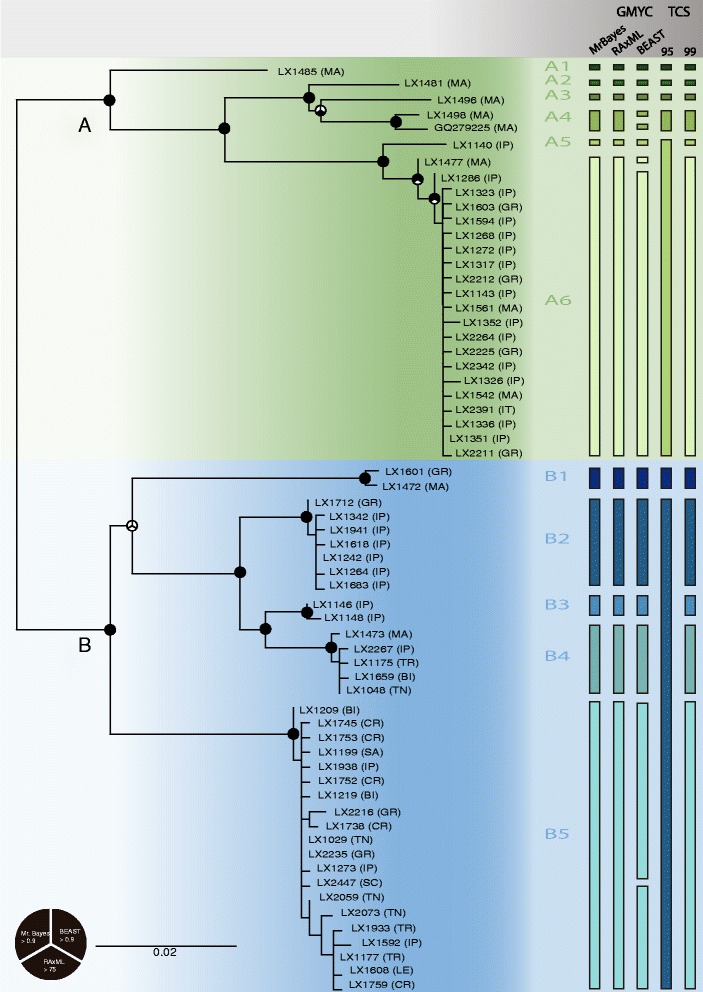
**Maximum likelihood tree based on single representatives of each** ***cox1*** **haplotype.** Node circles represent maximum likelihood bootstrap and Bayesian posterior probabilities, as shown in the legend. Green indicates the **A** clade, and blue the **B** clade. Each column on the right indicates a different delimitation method, and delimited lineages are represented with colored bars. Abbreviations: MA (Morocco), IP (Iberian Peninsula), GR (Greece), IT (Italian Peninsula), TR (Anatolian Peninsula), BI (Balearic Islands), TN (Tunisia), CR (Crete), SC (Sicily), LE (Levant).

Phylogenetic results were nearly identical between BI and ML approaches (available on TreeBase S15925). In both cases, *L. rufescens* was split in two main clades: A and B (Figure 3). Clade A included six well-supported lineages (all with bootstrap support >88% and posterior probabilities >0.76). Four lineages were composed exclusively of individuals from Morocco (termed lineages A1-A4). A1 placed as sister to a well-supported clade comprising A2-A6; the clade composed of A2-A4 placed as sister to a clade composed of A5-A6. A5 included individuals from two Iberian Peninsula populations, and A6 included individuals from across the Mediterranean.

Clade B comprised five lineages (B1-B5), all well-supported (bootstrap support >97%, posterior probabilities 1.0). B5 placed as sister to the remaining lineages of clade B, and lineage B1 was sister to a well-supported clade (bootstrap support 99%, posterior probabilities 1.0) comprising B2-B4, but this latter relationship was not well supported (bootstrap support 54%, posterior probabilities 0.64). B2 placed as sister to B3 and B4 (bootstrap support 79%, posterior probabilities 0.98). B3 was composed exclusively of Iberian Peninsula individuals; the remaining lineages included individuals from different Mediterranean regions.

Genetic p-distances ranged from 1.5-7.8% between the various lineages (Additional file 3). The two major clades (A and B) were separated by a p-distance of 7.04% (Additional file 3). Neutrality tests for the lineages are presented in Table 1. Fu’s *F*s test for demographic expansion was negative and significant in all cases except lineage B3. The *R*_2_ test was low and significant for the two lineages with higher sample sizes (A6 and B5) in the left tail. As a whole, these results suggest a recent demographic expansion for all lineages except for B3.

**Table 1:** **Summary of the nucleotide diversity estimates and neutrality tests**

**Lineage**	**n**	**Sites**	**H**	**H**_**D**_	**π**	***F***_**S**_	**95%** **CI**	***R***_**2**_	**95%** **CI**
A6	136	808	20	0.298	0.00045	−34.268***	−4.435-3.293	0.019*	0.015-0.238
B2	36	480	5	0.260	0.00057	−4.111***	−1.893-2.722	0.07 NS	0.054-0.25
B3	10	565	2	0.2	0.00035	−0.339 NS	−0.594-1.523	0.3 NS	0.178-0.3
B4	42	555	5	0.184	0.00043	−4.408***	−2.089-2.631	0.078 NS	0.046-0.254
B5	61	407	12	0.572	0.00187	−9.883***	−3.394-4.148	0.042*	0.046-0.23

*Abbreviations*: *p < 0.05; ***p < 0.01; *NS* non significant, *n* number of sequences, *H* number of haplotypes, *Hd* Haplotype diversity, *π* nucleotide diversity, *F*_S_ Fu's Fs, *R*_2_ Ramos-Onsins & Rozas *R*_2_ test, *CI* Confidence interval.

### Molecular delimitation analyses

We used three methods to obtain the ultrametric trees required for GMYC analyses (Figure 3). In all three cases, the likelihood ratio test of the Yule model was significantly better than the null hypothesis (BEAST *p* = 4.22 × 10^−6^, RAxML and PATHd8 *p* = 7.42 × 10^−7^, and MrBayes *p* = 1.28 × 10^−6^, respectively). Clusters identified (i.e. GMYC groups composed of more than single individuals) were mostly congruent across methods; in all three, seven clusters were delimited. Slight differences between approaches appeared in terms of detecting singletons: in total, we recovered 14 entities (clusters plus singletons) using the ultrametric tree obtained with BEAST (confidence interval: 11–21), while the remaining two analyses delimited 11 entities (confidence interval: 11–14 with RAxML, 10–11 with MrBayes). Differences occurred in lineages B5, A4, and A6, wherein GMYC analyses using the BEAST tree split each of these lineages into two clusters (Figure 3).

**Figure 4:**
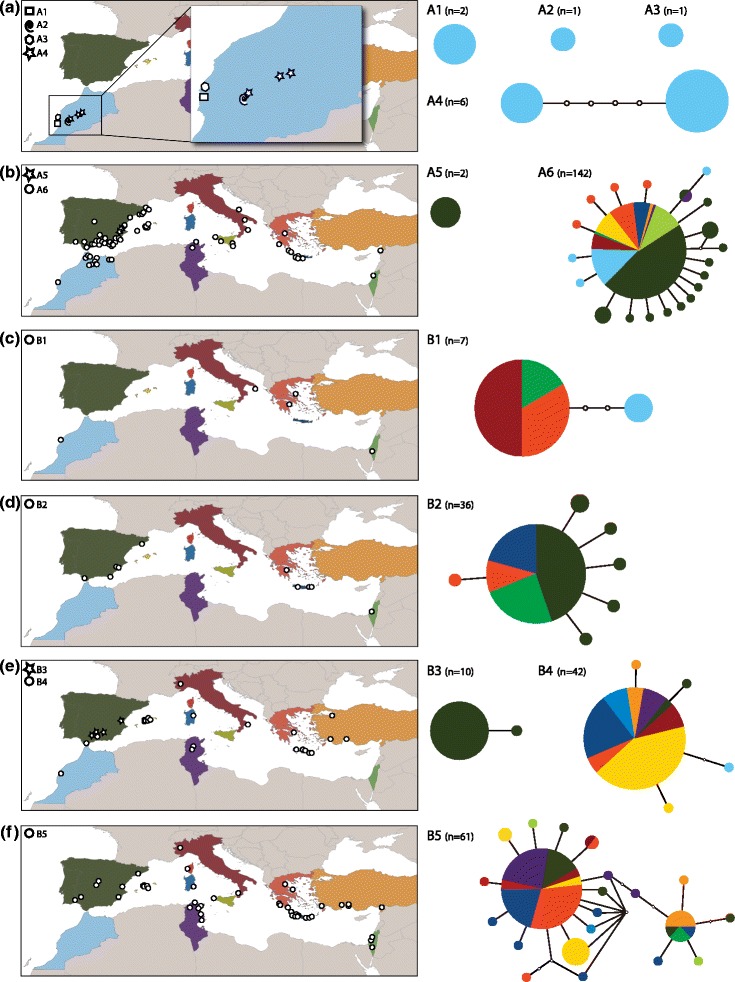
**Distribution map and haplotype network for each lineage.** Colors on haplotype networks correspond to colored areas on the maps to the left **(a-f)**. Haplotype networks are not to the same scale among lineages. Each circle represents one haplotype, and colors correspond to frequencies of region of origin for the haplotype (*n* = number of individuals). Note the two contrasting phylogeographic patterns, with lineages A1 to A5 restricted to one or a few well-structured populations, whereas the lineages distributed across the Mediterranean Basin generally lack geographic structure, which is likely a consequence of human-mediated dispersal.

TCS results varied depending on the connection limit (Figure 3): a 95% connection limit resulted in fewer independent networks compared to a 99% limit. In the former case, the maximum number of calculated steps was 13, forming seven independent networks, and in the latter case, the maximum number of calculated steps was five, with 11 independent networks. The higher connection limit mirrored the GMYC results. We found two main patterns in the haplotype networks: (1) several lineages composed of individuals restricted to one or a few localities that harbor only one or a few haplotypes, and conversely, (2) single haplotypes present in individuals from across the Mediterranean Basin, with closely related haplotypes forming a star-like network (Figure 4).

### Patterns of genetic diversity

Analyses were conducted across the entire dataset and within lineages with wide distributions (i.e. A6, B2, B4, and B5; results presented in Additional file 4). Analyses across all lineages lacked clear geographic patterns for total diversity (H_T_) and haplotype richness (H_R_); however, they exhibited higher values for the rarity index (R) in southern Morocco. Analyses of individual lineages showed clear geographic patterns for lineage B2 and B4, wherein higher values for all diversity statistics were obtained in the Iberian Peninsula and Balearic Islands, respectively. Lineages A6 and B5 showed no clear geographic pattern, with the highest values in various isolated regions. Different parametrizations for this test produced similar patterns (results not shown). That is, similar results were obtained when the number of individuals was reduced to three (not recommended by the script authors, N. Arrigo pers. comm.), except within lineage B2, where high values for all statistics characterized the region around Greece. The Mantel test showed weak correlations between genetic and geographic distance matrices for lineages A6, B2, B4, and B5 (*r* < 0.24), with tests not significant except for lineage B5 (Table 2). Conversely, when lineages A1-A5 were pooled, we obtained a stronger and significant correlation (*r* = 0.52, *P* < 0.05; Table 2), suggesting that they are geographically structured.

**Table 2:** **Results of Mantel tests performed with 10000 permutations to assess the significance of the correlation between genetic distances and log-transformed geographic distances**

**Lineage**	**r**	***P***
A1-A5	0.524	0.012
A6	0.058	0.176
B2	0.002	0.483
B4	0.159	0.059
B5	0.242	<0.0001

### Dating analyses

Divergence time estimates differed depending on the rate used in calibration (see Additional file 5). Both analyses, however, dated major diversification events to the period of Pleistocene glaciations. The estimated split between the two main clades (A *versus* B, Figure 3) was dated at 0.356 Ma (95% HPD: 0.243-0.487) using the *Loxosceles*-specific rate, and at 1.968 Ma (1.322-2.698) with the *Parachtes* rate.

### Niche models

#### Present

Models from individual AOGCMs were predictive of independent suites of occurrence points, with all models statistically significant in partial ROC tests (all *P* < 0.05; Additional file 6). Predictions were consistent across AOGCMs (Additional file 7), with suitable areas identified in the southern Iberian Peninsula, Italy, Greece, western Turkey, northern Africa, and various Mediterranean islands. Minor differences were noted among models; for example, less suitable area was identified in Turkey under MIROC and MRI. Regions environmentally outside those represented within **M** (highlighted by MOP analyses) were also consistent across AOGCMs, covering desert areas (e.g. the Sahara and Sinai Peninsula) and some regions around the Black Sea; these regions were not considered in our analyses.

#### Paleoprojections

Most regions identified as suitable in the present were also identified as suitable during the mid-Holocene (Additional file 7). MOP results were similar to those in the present, with one exception: although IPSL identified potential distributions congruent with those in other AOGCMs, MOP analyses indicated most of these regions were environmentally novel. A similar situation occurred with the LGM projection for MIROC. Refugial area delimitation was not affected by removal of these two models with odd results.

Compared to present and mid-Holocene projections, LGM potential distributions shifted southward. The main Mediterranean peninsulas (i.e. Iberia, Italy, Balkans) retained suitable conditions, although reduced in extent, but extensive regions of the Sahara, which were largely unsuitable in the present and mid-Holocene, were identified as suitable during the LGM under most AOGCMs. Regions environmentally outside those represented within **M** occurred across broad swaths of the northern and southeastern portions of our study area.

### Identifying refugial areas

**Figure 5:**
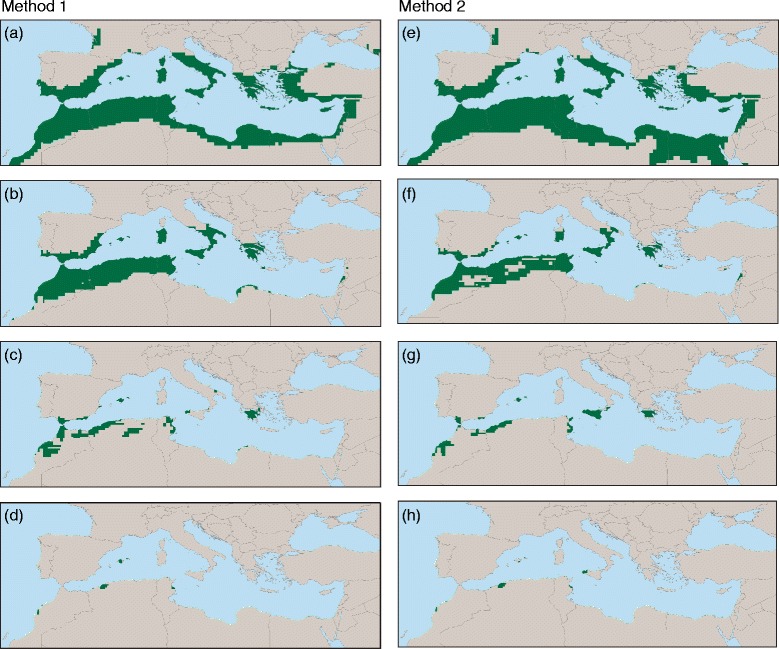
**Refugial maps developed via two delimitation methods.** Maps **a**, **b**, **c** and **d** were obtained with Method 1, and maps **e**, **f**, **g** and **h** were obtained using Method 2, as described in Figure 2.

Results from individual AOGCMs were similar, with the exception of IPSL. Because IPSL uses a different vegetation model from other AOGCMs (for example, bare soil is considered a type of vegetation, whereas other AOGCMs ignore this factor), we ran refugial delimitation analyses with and without IPSL. Putative refugia were congruent across the two methodologies and with and without IPSL (Figure 5; without IPSL not shown). Depending on the level of stringency enforced for AOGCM agreement, 4–14 major, independent, and isolated refugia were identified (Figure 5a-e). When less stringent agreement thresholds were applied (Figure 5a and e), the entire Mediterranean rim was identified as refugial, except for the northern and eastern coast of the Adriatic Sea and Gulf of Genoa. With intermediate levels of stringency (Figure 5b,c,f,g), most refugia were situated in the western Mediterranean, primarily Morocco, Algeria, and parts of the Iberian Peninsula (Cabo de Gata, Cadiz/Algeciras region, Valencia region; sensu Médail and Diadema [5]). The Balearic Islands, especially Mallorca, were identified as refugial, even under the most stringent criteria (Figure 5d and h), and some parts of Sicily were recovered under most scenarios (except the strictest criterion in M1). Unlike the western Mediterranean, few parts of the eastern half of the Mediterranean were identified as refugial. For example, only some areas of the Peloponnese (Greece) were recovered consistently as putative refugia; broad areas of Anatolia and the Levant coast were recovered as refugial only under the least stringent AOGCM agreement levels.

## Discussion

### Genetic diversity and biogeography

We document high mtDNA genetic diversity in *L. rufescens* across the Mediterranean Basin, as reported previously by Duncan et al. [32], underscoring the importance of broad sampling efforts for accurate representation of diversity patterns. Most of the delimitation methods recover 11 distinct evolutionary lineages (Figure 3), but molecular delimitation analyses, particularly those using only one line of evidence (in this case, mtDNA), can be prone to over-delimitation [90]. Thus, identified lineages should be taken as a basis for further studies of taxonomic status using integrative approaches based on morphology and variable nuclear markers [57], and the phylogeographic patterns uncovered herein merit reexamination with additional nuclear data. Nevertheless, even without further analyses, the existence of such divergent mitochondrial lineages deserves attention, and our main aim was to understand the factors promoting this diversity.

Genetic diversity is not distributed uniformly among lineages within *L. rufescens*, and is, in fact, highly heterogeneous (Figures 2 and 4 and Additional file 2). Broadly, we find two contrasting phylogeographic patterns: the mountainous region of Morocco harbors several lineages with well-structured populations, whereas lineages distributed across the broader Mediterranean Basin generally lack geographic structure.

Four lineages (A1-A4) are distributed along the western slopes of the Atlas Mountains (Figure 4), including individuals from A4 referred to as the “Asni clade” by Duncan et al. [32]. Although lineages A1-A4 live in close proximity (within 50–200 km), they exhibit striking genetic divergence (>4.7%, Additional file 3). The Atlas Mountain region shows the highest rarity index values in analyses considering all lineages (Additional file 4). This pattern of deep genetic divergence and haplotype differentiation among populations in close proximity may be explained by long-term presence of this species in the region and low dispersal capacity under natural conditions. Together with A5, these lineages are spatially structured in that genetic diversity is positively correlated with distance between localities. Altogether, these patterns are consistent with spider species with similar dispersal capacities (e.g. mygalomorphs, see [57]), and with other *Loxoscel*es species, such as those from the Canary Island [34], *Loxosce*l*es mra*z*i*g, and a related group from the Sous Valley (Morocco; Planas and Ribera unpublished data).

Most individuals, however, belong to lineages widespread across the Mediterranean (e.g. A6 and most B lineages, Figure 4). Haplotype networks for these lineages have star-like shapes, with one common haplotype shared among individuals distributed across the Mediterranean Basin, and fewer, less-common haplotypes, restricted to individuals from one or a few localities. For almost all lineages, no clear correspondence exists between haplotypes and geography, with weak correlations between genetic and geographic distances. For example, the most common haplotype from A6 is found across the entirety of the Mediterranean Basin (Figure 4). This lineage, named the “Iberian clade” by Duncan et al. [32], appears to be the most common in the western Mediterranean, and contains individuals from Sagunt, the type locality of *L. rufescens*.

Biogeographic patterns within clade B are more complex. The exception is lineage B3, where all individuals are found in the Iberian Peninsula. In lineages B2, B4 and B5, individuals with the most common haplotype are widespread across the Mediterranean, as in A6, although no clear pattern emerges that links genetic diversity with geography (Additional file 4 and Table 2). Lineage B1 represents the most extreme example of the complex distributional patterns found within *L. rufescens.* Even given the extensive sampling we conducted, we found individuals of this lineage at only five localities, some separated by >4000 km (although more samples from this lineage might produce the typical star-like shape of the B clade lineages).

Discerning between natural and human-mediated dispersals can be difficult in the Mediterranean ([91], and references herein). Current genetic patterns for Mediterranean lineages do not coincide with those expected as a result of secondary contact through natural processes. If naturally occurring, contact would be restricted to particular areas and/or occur between or among only a few lineages. Here, multiple lineages are distributed across the Mediterranean, including on islands, a pattern that is difficult to explain by natural processes in organisms with naturally poor dispersal abilities. The lack of geographic structure within most of the Mediterranean *Loxosceles* lineages contrasts with the highly structured patterns found for lineages A1-A4, distributed in the mountainous region of Morocco, with the former pattern a likely consequence of human-mediated dispersal. Although *L. rufescens* originated in the Mediterranean Basin ([30,32], see below), the species has been introduced to many parts of the world, including Australia, Madagascar and North America [30,32,34,92]. Human transportation seems a likely mechanism to explain how some haplotypes are distributed across the Mediterranean Basin, including on several islands and on both African and European shores. *Loxosceles rufescens* possesses two life traits that facilitate dispersal with human assistance: high starvation tolerance [93] and urban microhabitat preferences. Maritime commerce in this region has been active for >5000 years [2], and transportation of cultivated plants [94,95], domesticated animals [96], and wild animals such as reptiles [97], snails [98,99], mosquitoes [100], and freshwater triclads [101] has been documented widely throughout the Mediterranean. Thus, the expected “natural” biogeographic patterns have been blurred for this region, and the complex phylogeographic pattern documented for *L. rufescens* represents a clear example of human influence on species’ distributional dynamics.

### Refugia and origins of genetic diversity

Although human-mediated transportation of *L. rufescens* likely impacted patterns of distribution for this species within the Mediterranean Basin, the aim of this study was to assess when and how the distinct lineages originated. Combining divergence time and refugial estimates, we marshal two distinct data streams toward answering these questions [23].

Our dating analyses place diversification events during the Pleistocene (Additional file 5). Although we are not able to link diversification events to individual climatic events (i.e. a particular glacial-interglacial cycle) with any confidence, these dates provide a coarse-resolution estimate of diversification timing. The placement of key diversification events during the Pleistocene indicates that processes operating during this period (i.e. glacial/interglacial cycles) played an important role in shaping the current diversity of the species, as with numerous other species in the Mediterranean Basin (e.g. [98-106]). The results from our ENM analyses largely corroborate the scenario of multiple refugia within the Mediterranean for *L. rufescens*, although details depended on the level of stringency applied for agreement among the eight different AOGCM models (Figure 5). The shape and size of refugia differ markedly depending on the AOGCM used (Additional file 7). In other words, ENM is useful for providing general views of putative refugia, rather than for identifying actual borders. Combining results from different AOGCMs, we obtain a consensus view of general patterns for the latter half of the Pleistocene epoch [107], which, when an intermediate threshold (Figure 5) is considered, agrees with refugia obtained for plants using phylogeographic approaches [5].

In both methods (Figure 5), major refugia are concentrated in the western Maghreb. Indeed, in phylogenetic terms, four evolutionary lineages (A1-A4) are found in this area, signifying a hot spot of lineage richness. This richness supports the hypothesis that northern Africa is the center of origin for *L. rufescens*, as previously hypothesized by Gertch [30] and Duncan et al. [32]. Additionally, the sister group to *L. rufescens* is found south of this area, in the High and Anti-Atlas Mountains and the Sous Valley (Morocco; Planas and Ribera unpublished data), which lends further support to a northern African origin for these lineages and for *L. rufescens* as a whole. This region has been postulated as a climatic refugium for various animals and plants in light of its complex orography (e.g. Anti-Atlas, High Atlas) and climatic stability ([90] and references therein).

More challenging is linking putative refugia to the origins of the Mediterranean linages (A5, A6, B), with current distributional and genetic patterns most likely the result of population mixing through human-mediated transportation. Although some of these divergent lineages now occur in sympatry, they likely originated in allopatry, given the dominance of this speciation mechanism for the genus [31,34] and the geographic results summarized above (Figure 5). Lineages A5 and B3 have small distributions and are endemic to the Iberian Peninsula, which may reflect refugial areas, especially along the southern and eastern Iberian Mediterranean coast. However, five other lineages are widely distributed across the Mediterranean.

Genetic diversity patterns should help in elucidating the origins of these lineages, assuming that refugial areas harbor higher genetic diversity (but see [108]). Such is the case for lineage B4 (Additional file 4), where the highest genetic diversity is found in the Balearic Islands, an area identified consistently as a refugium (Figure 4 and Additional file 3). However, lineages A6, B1, B2, and B5 are widespread across the Mediterranean and do not show any correspondence between genetic diversity and a single putative refugial area; these lineages may have originated in one of the remaining predicted refugia (e.g. Sicily, southern Italian Peninsula, the Peloponnese), but subsequent processes (e.g. human-mediated transportation) appear to have erased ancient biogeographic signals. More extensive sampling in the central and eastern Mediterranean may help to resolve this question.

## Conclusions

In this study, we delimited 11 evolutionary lineages within *Loxosceles rufescens* in the Mediterranean Basin based on mtDNA data. Genetic diversity was not distributed uniformly, and we found two contrasting phylogeographic patterns: (1) the southern region of Morocco holds several lineages with well-structured populations, (2) whereas lineages distributed across the broader Mediterranean Basin generally lack geographic structure. By combining results from eight AOGCMs with two different refugium-delimitation methodologies, we corroborated the scenario of multiple refugia within the Mediterranean, principally in northern Africa. ENMs were useful for providing general views of putative refugia, with fine-scale details depending on the level of stringency applied for agreement among models. Although refugial delimitation remains challenging, by combining ENM with phylogeographic approaches, we found support for the hypothesis that northern Africa was the center of origin for *L. rufescens*, that current genetic diversity probably originated in allopatry and was promoted by successive glaciations during the Pleistocene, and that protracted human activities impacted the current distributional patterns of *L. rufescens* within the Mediterranean Basin.

## Availability of supporting data

The data sets supporting the phylogenetic results of this article are available on the TreeBase repository, ID: 15925, http://purl.org/phylo/treebase/phylows/study/TB2:S15925. Information on geographic location and Genbank accession number for the 310 individuals included in this study is available in Additional file 1.

## Additional files

Additional file 1:
**Information on specimens and Genbank accession numbers.** Information on geographic location, inclusion in ENM analysis (Y – yes; N – no), mtDNA lineage and Genbank accession number for the 310 individuals included in this study. In bold, individuals included in the phylogenetic analyses. Abbreviations: CS – Corsica; CR – Crete; GR - Greece, IB - Balearic Islands; IT – Italy; LE – Levant; MA - Morocco; PI – Iberian Peninsula; SA – Sardinia; SC – Sicily; TN – Tunisia; TR – Turkey. Additional file 2:
**Details on climatic models (AOGCMs) used for Ecological Niche Modeling.**
Additional file 3:
**Estimates of genetic p-distance between (above the diagonal) and within (in bold) lineages.** In the inset, genetic p-distance between the two clades. Additional file 4:
**Genetic diversity of** ***Loxosceles rufescens*** **in the Mediterranean.** Total diversity (HT), haplotype richness (HR) and rarity index (R) are represented for *L. rufescens* (all) and separately for lineages A6, B2, B4 and B5. Diversity statistics are computed by considering samples located within a perimeter around a grid point. We set grid points every 100 km in latitude and longitude, and computations were conducted across random sets of 5 individuals, bootstrapping 1000 times. Additional file 5:
**Dating analysis results.** Dating analysis results in Ma obtained from two different *cox1* rates: (1) using the rate for *Parachtes*, and (2) using a mean rate obtained for *Loxosceles*. Additional file 6:
**Partial ROC analyses.** Partial ROC tests (*P* < 0.05) using two data subsets with 1000 replicates. Additional file 7:
**Ecological niche modelling results for each atmosphere–ocean general circulation model (AOGCM).** Ecological niche modelling results for each AOGCM in each time slice (Last Glacial Maximum, mid-Holocene, and present).
