# Supplementary material for: Ecological niche and phylogeography elucidate complex biogeographic patterns in Loxosceles rufescens (Araneae, Sicariidae) in the Mediterranean Basin
Source: BMC Evol Biol. 2014 Oct 9;14:195. doi: 10.1186/s12862-014-0195-y (PMC4236462; doi:10.1186/s12862-014-0195-y)
Supplement: Additional file 1: — Information on specimens and Genbank accession numbers. Information on geographic location, inclusion in ENM analysis (Y – yes; N – no), mtDNA lineage and Genbank accession number for the 310 individuals included in this study. In bold, individuals included in the phylogenetic analyses. Abbreviations: CS – Corsica; CR – Crete; GR - Greece, IB - Balearic Islands; IT – Italy; LE – Levant; MA - Morocco; PI – Iberian Peninsula; SA – Sardinia; SC – Sicily; TN – Tunisia; TR – Turkey. [file 12862_2014_195_MOESM1_ESM.doc]

Additional file 1: Information on geographic location, inclusion in ENM analysis (Y – yes; N – no), mtDNA lineage and Genbank accession number for the 310 individuals included in this study. In bold, individuals included in the phylogenetic analyses. Abbreviations: CS – Corsica; CR – Crete; GR - Greece, IB - Balearic Islands; IT – Italy; LE – Levant; MA - Morocco; PI – Iberian Peninsula; SA – Sardinia; SC – Sicily; TN – Tunisia; TR – Turkey.

| ID | LONGITUDE | LATITUDE | ENM ANALYSIS | REGION | LINEAGE | *cox1*  ACCESSION NUMBER |
| --- | --- | --- | --- | --- | --- | --- |
| **LX1485_MA** | 31.15739 | -9.69767 | Y | MA | A1 | KJ560651 |
| LX1486_MA | 31.15739 | -9.69767 | Y | MA | A1 | KJ560652 |
| **LX1481_MA** | 31.50096 | -9.6158 | Y | MA | A2 | KJ560650 |
| **LX1496_MA** | 31.18955 | -8.05766 | Y | MA | A3 | KJ560653 |
| **GQ279225** | 31.250088 | -7.983352 | Y | MA | A4 | GQ279225 |
| GQ279226 | 31.250088 | -7.983352 | N | MA | A4 | GQ279226 |
| **LX1498_MA** | 31.95973 | -6.76811 | Y | MA | A4 | KJ560654 |
| LX1499_MA | 31.95973 | -6.76811 | Y | MA | A4 | KJ560655 |
| LX1507_MA | 32.08467 | -6.33358 | Y | MA | A4 | KJ560656 |
| LX1510_MA | 32.08467 | -6.33358 | Y | MA | A4 | KJ560657 |
| LX1348_PI | 38.86894 | -0.7589 | Y | PI | A5 | KJ560634 |
| **LX1140_PI** | 39.549464 | -0.41551 | N | PI | A5 | KJ560569 |
| LX1762_CR | 35.00651 | 24.94693 | Y | CR | A6 | KJ560747 |
| LX1763_CR | 35.00651 | 24.94693 | Y | CR | A6 | KJ560748 |
| LX1764_CR | 35.00651 | 24.94693 | Y | CR | A6 | KJ560749 |
| LX1735_CR | 35.1901167 | 24.44925 | Y | CR | A6 | KJ560733 |
| LX1746_CR | 35.2018667 | 24.16235 | Y | CR | A6 | KJ560739 |
| LX1741_CR | 35.32475 | 24.2812833 | Y | CR | A6 | KJ560736 |
| LX1742_CR | 35.32475 | 24.2812833 | Y | CR | A6 | KJ560737 |
| **LX2225_GR** | 35.881197 | 23.305596 | Y | GR | A6 | KJ560801 |
| LX2226_GR | 35.881197 | 23.305596 | Y | GR | A6 | KJ560802 |
| LX2227_GR | 35.88179 | 23.289468 | Y | GR | A6 | KJ560803 |
| LX2221_GR | 35.886535 | 23.295876 | Y | GR | A6 | KJ560799 |
| LX2222_GR | 35.886535 | 23.295876 | Y | GR | A6 | KJ560800 |
| LX2236_GR | 36.14744 | 23.018313 | Y | GR | A6 | KJ560807 |
| LX2237_GR | 36.14744 | 23.018313 | Y | GR | A6 | KJ560808 |
| LX2238_GR | 36.14744 | 23.018313 | Y | GR | A6 | KJ560809 |
| LX2239_GR | 36.14744 | 23.018313 | Y | GR | A6 | KJ560810 |
| LX2241_GR | 36.14744 | 23.018313 | Y | GR | A6 | KJ560812 |
| LX2242_GR | 36.14744 | 23.018313 | Y | GR | A6 | KJ560813 |
| **LX2211_GR** | 36.965 | 21.662 | Y | GR | A6 | KJ560796 |
| **LX2212_GR** | 36.965 | 21.662 | Y | GR | A6 | KJ560797 |
| **LX1603_GR** | 38.4523167 | 22.4213833 | Y | GR | A6 | KJ560686 |
| LX1591_IB | 38.94437 | 1.47775 | Y | IB | A6 | KJ560678 |
| LX1644_IB | 39.3943833 | 2.84726667 | Y | IB | A6 | KJ560708 |
| LX1633_IB | 39.4582333 | 2.76845 | Y | IB | A6 | KJ560698 |
| LX1638_IB | 39.4582333 | 2.76845 | Y | IB | A6 | KJ560702 |
| LX1640_IB | 39.4582333 | 2.76845 | Y | IB | A6 | KJ560704 |
| LX1650_IB | 39.4709667 | 3.0209 | Y | IB | A6 | KJ560709 |
| LX1673_IB | 39.5120333 | 2.92628333 | Y | IB | A6 | KJ560715 |
| LX1227_IB | 39.5979639 | 2.64130278 | Y | IB | A6 | KJ560598 |
| LX1390_IB | 39.8860972 | 3.06191944 | Y | IB | A6 | KJ560646 |
| **LX2391_IT** | 39.01587 | 17.16621 | Y | IT | A6 | KJ560844 |
| LX2383_IT | 40.46077 | 16.72798 | Y | IT | A6 | KJ560840 |
| LX2384_IT | 40.46077 | 16.72798 | Y | IT | A6 | KJ560841 |
| LX2385_IT | 40.46077 | 16.72798 | Y | IT | A6 | KJ560842 |
| LX2354_IT | 41.937 | 15.98725 | Y | IT | A6 | KJ560834 |
| LX2355_IT | 41.937 | 15.98725 | Y | IT | A6 | KJ560835 |
| LX2356_IT | 41.937 | 15.98725 | Y | IT | A6 | KJ560836 |
| LX1834_LE | 32.535773 | 34.949127 | N | LE | A6 | KJ560767 |
| **LX1477_MA** | 31.60901 | -9.65915 | Y | MA | A6 | KJ560649 |
| LX1532_MA | 34.0573 | -4.23691 | Y | MA | A6 | KJ560661 |
| LX1538_MA | 34.0573 | -4.23691 | Y | MA | A6 | KJ560662 |
| LX1520_MA | 34.05933 | -5.30338 | Y | MA | A6 | KJ560658 |
| LX1523_MA | 34.10654 | -4.96948 | Y | MA | A6 | KJ560659 |
| LX1530_MA | 34.12708 | -4.30482 | Y | MA | A6 | KJ560660 |
| LX1577_MA | 34.5612 | -4.49946 | Y | MA | A6 | KJ560669 |
| LX1550_MA | 34.80333 | -2.39686 | Y | MA | A6 | KJ560664 |
| **LX1561_MA** | 34.80333 | -2.39686 | Y | MA | A6 | KJ560665 |
| **LX1542_MA** | 34.82454 | -2.08165 | Y | MA | A6 | KJ560663 |
| LX1564_MA | 34.83786 | -2.35774 | Y | MA | A6 | KJ560666 |
| LX1567_MA | 34.83786 | -2.35774 | Y | MA | A6 | KJ560667 |
| LX1571_MA | 35.00546 | -4.17899 | Y | MA | A6 | KJ560668 |
| LX1578_MA | 35.00546 | -4.17899 | Y | MA | A6 | KJ560670 |
| LX1136_MA | 35.0868738 | -5.7641125 | Y | MA | A6 | KJ560568 |
| LX1134_MA | 35.2380333 | -5.1740167 | Y | MA | A6 | KJ560566 |
| LX1135_MA | 35.2380333 | -5.1740167 | Y | MA | A6 | KJ560567 |
| LX1377_MA | 35.3639073 | -5.3715844 | Y | MA | A6 | KJ560643 |
| LX1944_PI | 36.51784 | -5.65624 | Y | PI | A6 | KJ560782 |
| LX1334_PI | 36.80214 | -2.14298 | Y | PI | A6 | KJ560630 |
| LX1335_PI | 36.80214 | -2.14298 | Y | PI | A6 | KJ560631 |
| **LX1336_PI** | 36.80214 | -2.14298 | Y | PI | A6 | KJ560632 |
| **LX1326_PI** | 36.84535 | -2.01746 | Y | PI | A6 | KJ560627 |
| LX1327_PI | 36.84535 | -2.01746 | Y | PI | A6 | KJ560628 |
| **LX1317_PI** | 36.84788 | -2.0249 | Y | PI | A6 | KJ560623 |
| LX1319_PI | 36.84788 | -2.0249 | Y | PI | A6 | KJ560624 |
| **LX1323_PI** | 36.84788 | -2.0249 | Y | PI | A6 | KJ560625 |
| LX1325_PI | 36.84788 | -2.0249 | Y | PI | A6 | KJ560626 |
| LX1315_PI | 36.94333 | -1.90926 | Y | PI | A6 | KJ560622 |
| LX2279_PI | 36.9617 | -3.8662 | Y | PI | A6 | KJ560825 |
| LX2456_PI | 36.970732 | -4.832509 | Y | PI | A6 | KJ560860 |
| LX1305_PI | 36.98014 | -2.17114 | Y | PI | A6 | KJ560619 |
| LX1306_PI | 36.98014 | -2.17114 | Y | PI | A6 | KJ560620 |
| LX1313_PI | 36.98014 | -2.17114 | Y | PI | A6 | KJ560621 |
| **LX1014_PI** | 36.99055 | -2.399166 | Y | PI | A6 | KJ560560 |
| LX2283_PI | 37.0361522 | -4.4987515 | N | PI | A6 | KJ560828 |
| LX2287_PI | 37.0361522 | -4.4987515 | N | PI | A6 | KJ560829 |
| LX1299_PI | 37.04982 | -2.20497 | Y | PI | A6 | KJ560617 |
| LX1300_PI | 37.04982 | -2.20497 | Y | PI | A6 | KJ560618 |
| LX2277_PI | 37.17809 | -3.05636 | Y | PI | A6 | KJ560823 |
| LX2278_PI | 37.17809 | -3.05636 | Y | PI | A6 | KJ560824 |
| **LX1286_PI** | 37.21327 | -1.82724 | Y | PI | A6 | KJ560613 |
| LX1288_PI | 37.21327 | -1.82724 | Y | PI | A6 | KJ560614 |
| LX1940_PI | 37.25949 | -7.2 | Y | PI | A6 | KJ560780 |
| LX1290_PI | 37.29408 | -2.22548 | Y | PI | A6 | KJ560615 |
| LX1291_PI | 37.29408 | -2.22548 | Y | PI | A6 | KJ560616 |
| LX1279_PI | 37.32173 | -1.70208 | Y | PI | A6 | KJ560612 |
| LX1606_PI | 37.36468 | -3.47183 | Y | PI | A6 | KJ560689 |
| LX1196_PI | 37.3658056 | -3.46925 | Y | PI | A6 | KJ560586 |
| LX2263_PI | 37.385707 | -5.981608 | N | PI | A6 | KJ560818 |
| **LX1594_PI** | 37.4763427 | -4.2792721 | Y | PI | A6 | KJ560680 |
| LX1605_PI | 37.49108 | -3.82442 | Y | PI | A6 | KJ560688 |
| LX1276_PI | 37.6079 | -0.75988 | Y | PI | A6 | KJ560610 |
| LX1277_PI | 37.60821 | -0.75619 | Y | PI | A6 | KJ560611 |
| LX2282_PI | 37.85253 | -1.47114 | Y | PI | A6 | KJ560827 |
| LX1267_PI | 37.92965 | -1.13036 | Y | PI | A6 | KJ560604 |
| **LX1268_PI** | 37.92965 | -1.13036 | Y | PI | A6 | KJ560605 |
| LX1270_PI | 37.92965 | -1.13036 | Y | PI | A6 | KJ560606 |
| **LX1272_PI** | 37.92965 | -1.13036 | Y | PI | A6 | KJ560607 |
| LX1275_PI | 37.92965 | -1.13036 | Y | PI | A6 | KJ560609 |
| LX1190_PI | 37.9984214 | -2.9874861 | N | PI | A6 | KJ560584 |
| LX2269_PI | 38.36102 | -3.75513 | Y | PI | A6 | KJ560821 |
| LX1356_PI | 39.54699 | -0.5102 | Y | PI | A6 | KJ560637 |
| LX1359_PI | 39.54699 | -0.5102 | Y | PI | A6 | KJ560638 |
| LX1141_PI | 39.549464 | -0.41551 | N | PI | A6 | KJ560570 |
| LX1142_PI | 39.549464 | -0.41551 | N | PI | A6 | KJ560571 |
| **LX1143_PI** | 39.549464 | -0.41551 | N | PI | A6 | KJ560572 |
| LX1144_PI | 39.549464 | -0.41551 | N | PI | A6 | KJ560573 |
| LX1145_PI | 39.549464 | -0.41551 | N | PI | A6 | KJ560574 |
| LX1874_PI | 39.6678667 | -0.2886833 | Y | PI | A6 | KJ560774 |
| **LX1351_PI** | 39.68481 | -0.30005 | Y | PI | A6 | KF717003 |
| **LX1352_PI** | 39.68481 | -0.30005 | Y | PI | A6 | KJ560635 |
| LX1354_PI | 39.68481 | -0.30005 | Y | PI | A6 | KJ560636 |
| LX2454_PI | 40.30466 | -4.61335 | Y | PI | A6 | KJ560858 |
| LX2455_PI | 40.30466 | -4.61335 | Y | PI | A6 | KJ560859 |
| LX1822_PI | 40.86461 | 0.501121 | Y | PI | A6 | KJ560760 |
| **LX2342_PI** | 40.86461 | 0.501121 | Y | PI | A6 | KJ560830 |
| LX2347_PI | 40.86461 | 0.501121 | Y | PI | A6 | KJ560831 |
| LX2348_PI | 40.86461 | 0.501121 | Y | PI | A6 | KJ560832 |
| LX2349_PI | 40.86461 | 0.501121 | Y | PI | A6 | KJ560833 |
| LX2280_PI | 41.282 | 1.835 | Y | PI | A6 | KJ560826 |
| LX2151_PI | 41.38605 | 2.1639 | N | PI | A6 | KJ560793 |
| LX2152_PI | 41.38605 | 2.1639 | N | PI | A6 | KJ560794 |
| **LX2264_PI** | 41.38605 | 2.1639 | N | PI | A6 | KJ560819 |
| LX2458_PI | 41.640269 | 2.403385 | Y | PI | A6 | KJ560862 |
| LX2459_PI | 41.640269 | 2.403385 | Y | PI | A6 | KJ560863 |
| LX1191_PI | 41.9181333 | 3.1639 | Y | PI | A6 | KJ560585 |
| LX1823_PI | 41.9181333 | 3.1639 | Y | PI | A6 | KJ560761 |
| LX1590_PI | 42.2439458 | 2.694298 | Y | PI | A6 | KJ560677 |
| LX1819_SC | 36.72157 | 15.11847 | Y | SC | A6 | KJ560757 |
| LX1820_SC | 36.72157 | 15.11847 | Y | SC | A6 | KJ560758 |
| LX1821_SC | 36.72157 | 15.11847 | Y | SC | A6 | KJ560759 |
| LX2411_SC | 36.72166 | 15.11794 | Y | SC | A6 | KJ560847 |
| LX2412_SC | 36.72166 | 15.11794 | Y | SC | A6 | KJ560848 |
| LX2413_SC | 36.72166 | 15.11794 | Y | SC | A6 | KJ560849 |
| LX2420_SC | 37.13928 | 15.03244 | Y | SC | A6 | KJ560850 |
| LX2423_SC | 37.13928 | 15.03244 | Y | SC | A6 | KJ560851 |
| LX2424_SC | 37.13928 | 15.03244 | Y | SC | A6 | KJ560852 |
| LX2434_SC | 37.39064 | 13.29294 | Y | SC | A6 | KJ560853 |
| LX2436_SC | 37.39064 | 13.29294 | Y | SC | A6 | KJ560854 |
| LX2448_SC | 38.08555 | 12.67299 | Y | SC | A6 | KJ560856 |
| LX1046_TN | 36.5300442 | 9.36006146 | Y | TN | A6 | FJ986186 |
| LX2084_TN | 37.329 | 9.84616 | Y | TN | A6 | KJ560788 |
| LX1178_TR | 36.1113889 | 35.9458333 | Y | TR | A6 | KJ560583 |
| LX1714_GR | 37.7224667 | 22.7517 | Y | GR | B1 | KJ560726 |
| **LX1601_GR** | 39.1923333 | 23.9233333 | Y | GR | B1 | KJ560684 |
| LX2369_IT | 39.86921 | 18.24284 | Y | IT | B1 | KJ560837 |
| LX2370_IT | 39.86921 | 18.24284 | Y | IT | B1 | KJ560838 |
| LX2371_IT | 39.86921 | 18.24284 | Y | IT | B1 | KJ560839 |
| LX1609_LE | 30.897868 | 34.894342 | Y | LE | B1 | KJ560692 |
| **LX1472_MA** | 32.50405 | -9.25307 | Y | MA | B1 | KJ560647 |
| LX1584_CR | 35.215925 | 26.0701278 | Y | CR | B2 | KJ560675 |
| LX1582_CR | 35.2188889 | 26.0690722 | Y | CR | B2 | KJ560673 |
| LX1583_CR | 35.2188889 | 26.0690722 | Y | CR | B2 | KJ560674 |
| LX1770_CR | 35.25165 | 26.25206 | Y | CR | B2 | KJ560754 |
| LX1771_CR | 35.25165 | 26.25206 | Y | CR | B2 | KJ560755 |
| LX1737_CR | 35.3508167 | 24.35395 | Y | CR | B2 | KJ560734 |
| **LX1712_GR** | 37.7224667 | 22.7517 | Y | GR | B2 | KJ560724 |
| LX1713_GR | 37.7224667 | 22.7517 | Y | GR | B2 | KJ560725 |
| LX1715_GR | 37.7224667 | 22.7517 | Y | GR | B2 | KJ560727 |
| LX1716_GR | 37.7224667 | 22.7517 | Y | GR | B2 | KJ560728 |
| LX1829_LE | 32.116533 | 34.799977 | N | LE | B2 | KJ560762 |
| LX1830_LE | 32.116533 | 34.799977 | N | LE | B2 | KJ560763 |
| LX1831_LE | 32.116533 | 34.799977 | N | LE | B2 | KJ560764 |
| LX1832_LE | 32.116533 | 34.799977 | N | LE | B2 | KJ560765 |
| LX1835_LE | 32.116533 | 34.799977 | N | LE | B2 | KJ560768 |
| LX1837_LE | 32.116533 | 34.799977 | N | LE | B2 | KJ560770 |
| LX1838_LE | 32.116533 | 34.799977 | N | LE | B2 | KJ560771 |
| **LX1941_PI** | 36.44859 | -5.88949 | Y | PI | B2 | KJ560781 |
| LX1330_PI | 36.7285 | -2.19096 | Y | PI | B2 | KF717002 |
| LX1331_PI | 36.7285 | -2.19096 | Y | PI | B2 | KJ560629 |
| **LX1342_PI** | 36.80214 | -2.14298 | Y | PI | B2 | KJ560633 |
| **LX1242_PI** | 38.0362 | -1.09445 | Y | PI | B2 | KJ560600 |
| LX1244_PI | 38.0362 | -1.09445 | Y | PI | B2 | KJ560601 |
| **LX1264_PI** | 38.0362 | -1.09445 | Y | PI | B2 | KJ560602 |
| **LX1618_PI** | 38.0362 | -1.09445 | Y | PI | B2 | KJ560695 |
| LX1620_PI | 38.0362 | -1.09445 | Y | PI | B2 | KJ560696 |
| LX1630_PI | 38.0362 | -1.09445 | Y | PI | B2 | KJ560697 |
| LX1680_PI | 38.0362 | -1.09445 | Y | PI | B2 | KJ560717 |
| LX1681_PI | 38.0362 | -1.09445 | Y | PI | B2 | KJ560718 |
| LX1682_PI | 38.0362 | -1.09445 | Y | PI | B2 | KJ560719 |
| **LX1683_PI** | 38.0362 | -1.09445 | Y | PI | B2 | KJ560720 |
| LX1684_PI | 38.0362 | -1.09445 | Y | PI | B2 | KJ560721 |
| LX1685_PI | 38.0362 | -1.09445 | Y | PI | B2 | KJ560722 |
| LX1686_PI | 38.0362 | -1.09445 | Y | PI | B2 | KJ560723 |
| LX1607_PI | 38.08524 | -1.36514 | Y | PI | B2 | KJ560690 |
| LX2457_PI | 41.640269 | 2.403385 | Y | PI | B2 | KJ560861 |
| **LX1146_PI** | 37.383717 | -4.786571 | N | PI | B3 | KJ560575 |
| LX1596_PI | 37.4958902 | -4.2559177 | Y | PI | B3 | KJ560681 |
| LX2271_PI | 37.79839 | -3.08411 | Y | PI | B3 | KJ560822 |
| LX1361_PI | 39.53622 | -0.62619 | Y | PI | B3 | KJ560639 |
| LX1365_PI | 39.53622 | -0.62619 | Y | PI | B3 | KJ560640 |
| LX1369_PI | 39.53622 | -0.62619 | Y | PI | B3 | KJ560641 |
| LX1375_PI | 39.53622 | -0.62619 | Y | PI | B3 | KJ560642 |
| LX1376_PI | 39.53622 | -0.62619 | Y | PI | B3 | KF717004 |
| LX1860_PI | 39.53622 | -0.62619 | Y | PI | B3 | KJ560772 |
| **LX1148_PI** | 39.536261 | -0.6263104 | Y | PI | B3 | KJ560576 |
| LX1767_CR | 35.04143 | 26.19772 | Y | CR | B4 | KJ560751 |
| LX1768_CR | 35.04143 | 26.19772 | Y | CR | B4 | KJ560752 |
| LX1747_CR | 35.2018667 | 24.16235 | Y | CR | B4 | KJ560740 |
| LX1769_CR | 35.25165 | 26.25206 | Y | CR | B4 | KJ560753 |
| LX1766_CR | 35.26239 | 25.38262 | Y | CR | B4 | KJ560750 |
| LX1728_CR | 35.3917333 | 25.0268833 | Y | CR | B4 | KJ560729 |
| LX1729_CR | 35.3917333 | 25.0268833 | Y | CR | B4 | KJ560730 |
| LX1730_CR | 35.3917333 | 25.0268833 | Y | CR | B4 | KJ560731 |
| LX1585_GR | 37.9254833 | 23.7584667 | Y | GR | B4 | KJ560676 |
| LX1604_GR | 37.9254833 | 23.7584667 | Y | GR | B4 | KJ560687 |
| LX1210_IB | 39.3468472 | 3.18578889 | Y | IB | B4 | KJ560592 |
| LX1635_IB | 39.4582333 | 2.76845 | Y | IB | B4 | KJ560699 |
| LX1636_IB | 39.4582333 | 2.76845 | Y | IB | B4 | KJ560700 |
| LX1637_IB | 39.4582333 | 2.76845 | Y | IB | B4 | KJ560701 |
| LX1639_IB | 39.4582333 | 2.76845 | Y | IB | B4 | KJ560703 |
| LX1641_IB | 39.4582333 | 2.76845 | Y | IB | B4 | KJ560705 |
| LX1642_IB | 39.4582333 | 2.76845 | Y | IB | B4 | KJ560706 |
| LX1643_IB | 39.4582333 | 2.76845 | Y | IB | B4 | KJ560707 |
| **LX1659_IB** | 39.5333 | 2.53353333 | Y | IB | B4 | KJ560711 |
| LX1612_IB | 39.5642139 | 2.54599444 | Y | IB | B4 | KJ560693 |
| LX1229_IB | 39.5643778 | 2.54600556 | Y | IB | B4 | KJ560599 |
| LX1212_IB | 39.6059808 | 2.59872944 | Y | IB | B4 | KJ560594 |
| LX1213_IB | 39.6059808 | 2.59872944 | Y | IB | B4 | KJ560595 |
| LX1669_IB | 39.68175 | 2.55013333 | Y | IB | B4 | KJ560713 |
| LX1655_IB | 39.7392667 | 3.42635 | Y | IB | B4 | KJ560710 |
| LX1671_IB | 39.7480833 | 3.07003333 | Y | IB | B4 | KJ560714 |
| LX1388_IB | 39.9156694 | 3.07403333 | Y | IB | B4 | KJ560645 |
| LX2390_IT | 39.01587 | 17.16621 | Y | IT | B4 | KJ560843 |
| LX2395_IT | 39.01587 | 17.16621 | Y | IT | B4 | KJ560845 |
| LX1131_IT | 45.054061 | 7.702051 | N | IT | B4 | KJ560565 |
| **LX1473_MA** | 31.9564 | -9.31604 | Y | MA | B4 | KJ560648 |
| **LX2267_PI** | 36.38845 | -5.65146 | Y | PI | B4 | KJ560820 |
| LX1597_PI | 37.476554 | -4.1728106 | Y | PI | B4 | KJ560682 |
| LX1200_SA | 40.2886111 | 9.50725 | Y | SA | B4 | KJ560588 |
| LX1202_SA | 40.2886111 | 9.50725 | Y | SA | B4 | KJ560589 |
| LX1207_SA | 40.2886111 | 9.50725 | Y | SA | B4 | KJ560590 |
| LX2095_TN | 35.47567 | 9.34092 | Y | TN | B4 | KJ560789 |
| LX2097_TN | 35.47567 | 9.34092 | Y | TN | B4 | KJ560790 |
| **LX1048_TN** | 35.9165506 | 9.55883798 | Y | TN | B4 | KJ560562 |
| **LX1175_TR** | 36.938501 | 31.169117 | N | TR | B4 | KJ560580 |
| LX1174_TR | 36.973232 | 28.916784 | N | TR | B4 | KJ560579 |
| LX1173_TR | 40.265756 | 29.121893 | N | TR | B4 | KJ560578 |
| LX1580_CR | 35.0381306 | 25.4583583 | Y | CR | B5 | KJ560671 |
| LX1581_CR | 35.0479444 | 26.0101694 | Y | CR | B5 | KJ560672 |
| LX1734_CR | 35.1901167 | 24.44925 | Y | CR | B5 | KJ560732 |
| **LX1745_CR** | 35.2018667 | 24.16235 | Y | CR | B5 | KJ560738 |
| LX1772_CR | 35.25165 | 26.25206 | Y | CR | B5 | KJ560756 |
| **LX1759_CR** | 35.3273167 | 23.5537833 | Y | CR | B5 | KJ560745 |
| LX1760_CR | 35.3273167 | 23.5537833 | Y | CR | B5 | KJ560746 |
| **LX1738_CR** | 35.3508167 | 24.35395 | Y | CR | B5 | KJ560735 |
| LX1749_CR | 35.43195 | 23.94625 | Y | CR | B5 | KJ560741 |
| LX1750_CR | 35.43195 | 23.94625 | Y | CR | B5 | KJ560742 |
| **LX1752_CR** | 35.51075 | 24.06835 | Y | CR | B5 | KJ560743 |
| **LX1753_CR** | 35.51075 | 24.06835 | Y | CR | B5 | KJ560744 |
| LX1920_CS | 42.01999 | 8.72525 | Y | CS | B5 | KJ560775 |
| LX2232_GR | 36.14744 | 23.018313 | Y | GR | B5 | KJ560804 |
| LX2233_GR | 36.14744 | 23.018313 | Y | GR | B5 | KJ560805 |
| **LX2235_GR** | 36.14744 | 23.018313 | Y | GR | B5 | KJ560806 |
| LX2240_GR | 36.14744 | 23.018313 | Y | GR | B5 | KJ560811 |
| LX2246_GR | 36.14744 | 23.018313 | Y | GR | B5 | KJ560814 |
| LX2247_GR | 36.14744 | 23.018313 | Y | GR | B5 | KJ560815 |
| LX2260_GR | 36.480069 | 22.967564 | Y | GR | B5 | KJ560816 |
| LX2261_GR | 36.480069 | 22.967564 | Y | GR | B5 | KJ560817 |
| LX2200_GR | 36.82893 | 21.87662 | Y | GR | B5 | KJ560795 |
| **LX2216_GR** | 36.965 | 21.662 | Y | GR | B5 | KJ560798 |
| LX1602_GR | 39.0907861 | 23.6605722 | Y | GR | B5 | KJ560685 |
| LX1600_GR | 39.85435 | 22.5398333 | Y | GR | B5 | KJ560683 |
| **LX1209_IB** | 39.3468472 | 3.18578889 | Y | IB | B5 | KJ560591 |
| LX1211_IB | 39.3468472 | 3.18578889 | Y | IB | B5 | KJ560593 |
| LX1660_IB | 39.5333 | 2.53353333 | Y | IB | B5 | KJ560712 |
| LX1676_IB | 39.5826167 | 3.01868333 | Y | IB | B5 | KJ560716 |
| LX1613_IB | 39.597975 | 2.64130278 | Y | IB | B5 | KJ560694 |
| **LX1219_IB** | 39.609295 | 2.5991 | Y | IB | B5 | KJ560596 |
| LX1220_IB | 39.609295 | 2.5991 | Y | IB | B5 | KJ560597 |
| LX2404_IT | 38.58894 | 15.89409 | Y | IT | B5 | KJ560846 |
| LX1129_IT | 45.054061 | 7.702051 | N | IT | B5 | KJ560564 |
| **LX1608_LE** | 30.897868 | 34.894342 | Y | LE | B5 | KJ560691 |
| LX1836_LE | 32.116533 | 34.799977 | N | LE | B5 | KJ560769 |
| LX1833_LE | 32.535773 | 34.949127 | N | LE | B5 | KJ560766 |
| **LX1938_PI** | 37.61826 | -7.2 | Y | PI | B5 | KJ560779 |
| LX1266_PI | 37.92965 | -1.13036 | Y | PI | B5 | KJ560603 |
| **LX1273_PI** | 37.92965 | -1.13036 | Y | PI | B5 | KJ560608 |
| **LX1592_PI** | 37.99675 | -6.3422 | Y | PI | B5 | KJ560679 |
| LX1862_PI | 39.5660667 | -0.4582333 | Y | PI | B5 | KJ560773 |
| LX2453_PI | 39.75239 | -4.21469 | Y | PI | B5 | KJ560857 |
| LX1058_PI | 40.318501 | -3.880352 | N | PI | B5 | KJ560563 |
| LX1385_PI | 41.3922222 | 1.80777778 | Y | PI | B5 | KJ560644 |
| **LX1199_SA** | 39.5305 | 9.59586111 | Y | SA | B5 | KJ560587 |
| **LX2447_SC** | 38.08555 | 12.67299 | Y | SC | B5 | KJ560855 |
| LX1151_TN | 35.6201759 | 10.6005232 | Y | TN | B5 | KJ560577 |
| LX2142_TN | 36.46527 | 10.27901 | Y | TN | B5 | KJ560791 |
| LX2143_TN | 36.46527 | 10.27901 | Y | TN | B5 | KJ560792 |
| **LX1029_TN** | 36.5101533 | 9.13655918 | Y | TN | B5 | KJ560561 |
| LX2058_TN | 36.77661 | 10.58439 | Y | TN | B5 | KJ560783 |
| **LX2059_TN** | 36.77661 | 10.58439 | Y | TN | B5 | KJ560784 |
| LX2067_TN | 36.77661 | 10.58439 | Y | TN | B5 | KJ560785 |
| **LX2073_TN** | 37.329 | 9.84616 | Y | TN | B5 | KJ560786 |
| LX2081_TN | 37.329 | 9.84616 | Y | TN | B5 | KJ560787 |
| LX1176_TR | 36.508913 | 36.186102 | N | TR | B5 | KJ560581 |
| LX1935_TR | 36.726286 | 27.689188 | N | TR | B5 | KJ560778 |
| **LX1933_TR** | 36.777248 | 31.475971 | N | TR | B5 | KJ560776 |
| **LX1177_TR** | 36.9716667 | 31.5333333 | Y | TR | B5 | KJ560582 |
| LX1934_TR | 36.974331 | 30.573129 | N | TR | B5 | KJ560777 |
