# Supplementary material for: Ecological niche and phylogeography elucidate complex biogeographic patterns in Loxosceles rufescens (Araneae, Sicariidae) in the Mediterranean Basin
Source: BMC Evol Biol. 2014 Oct 9;14:195. doi: 10.1186/s12862-014-0195-y (PMC4236462; doi:10.1186/s12862-014-0195-y)
Supplement: Additional file 2: — Details on climatic models (AOGCMs) used for Ecological Niche Modeling. [file 12862_2014_195_MOESM2_ESM.doc]

Additional file 2: Details on climatic models (AOGCMs) used for Ecological Niche Modeling.

| **Model ID** | **Modeling Center** | **Resolution*** | **Source** | **Year** |
| --- | --- | --- | --- | --- |
| CCSM4 | National Center for Atmospheric Research, USA | 0.9° × 1.25° | CMIP5/PMIP3 | 2012 |
| CNRM-CM5 | Centre National de Recherches Meteorologiques / Centre Europeen de Recherche et Formation Avancees en Calcul Scientifique, France | 1.4° x 1.4° | CMIP5/PMIP3 | 2012 |
| COSMOS-ASO (FUB) | Freie Universität Berlin, Germany | 3.75 x 3.7 | PMIP3 | 2012 |
| GISS-E2-R | NASA Goddard Institute for Space Studies, USA | 2.5° x 2.0° | CMIP5/PMIP3 | 2012 |
| MIROC-ESM | Atmosphere and Ocean Research Institute (University of Tokyo), National Institute for Environmental Studies, and Japan Agency for Marine-Earth Science and Technology, Japan | 2.8° × 2.8° | CMIP5/PMIP3 | 2012 |
| IPSL-CM5A-LR | Institut Pierre Simon Laplace, France | 3.75 x 1.9 | CMIP5/PMIP3 | 2012 |
| MPI-ESM-P | Max Planck Institute for Meteorology, Germany | 1.9 x 1.9 | CMIP5/PMIP3 | 2011 |
| MRI-CGCM3 | Meteorological Research Institute, Japan | 1.1° x 1.1° | CMIP5/PMIP3 | 2012 |

* Longitude × latitude

CMIP5 – Coupled Model Intercomparison Project, Phase 5 (<http://cmip-pcmdi.llnl.gov/>)

PMIP3 – Paleoclimate Modelling Intercomparison Project, Phase 3 (<http://pmip3.lsce.ipsl.fr/>
