# Supplementary material for: Ecological niche and phylogeography elucidate complex biogeographic patterns in Loxosceles rufescens (Araneae, Sicariidae) in the Mediterranean Basin
Source: BMC Evol Biol. 2014 Oct 9;14:195. doi: 10.1186/s12862-014-0195-y (PMC4236462; doi:10.1186/s12862-014-0195-y)
Supplement: Additional file 3: — Estimates of genetic p-distance between (above the diagonal) and within (in bold) lineages. In the inset, genetic p-distance between the two clades. [file 12862_2014_195_MOESM3_ESM.doc]

Additional file 3: Estimates of genetic p-distance between (above the diagonal) and within (in bold) lineages. In the inset genetic p-distance between the two clades.

|  | A1 | A2 | A3 | A4 | A5 | A6 | B1 | B2 | B3 | B4 | B5 |
| --- | --- | --- | --- | --- | --- | --- | --- | --- | --- | --- | --- |
| A1 | **0.000** |  |  |  |  |  |  |  |  |  |  |
| A2 | 0.047 | **-** |  |  |  |  |  |  | Clade | B |  |
| A3 | 0.055 | 0.026 | **-** |  |  |  |  |  | A | 0.07 |  |
| A4 | 0.051 | 0.024 | 0.025 | **0.004** |  |  |  |  |  |  |  |
| A5 | 0.047 | 0.041 | 0.047 | 0.042 | **0.000** |  |  |  |  |  |  |
| A6 | 0.048 | 0.041 | 0.048 | 0.042 | 0.015 | **0.000** |  |  |  |  |  |
| B1 | 0.059 | 0.071 | 0.078 | 0.069 | 0.075 | 0.076 | **0.001** |  |  |  |  |
| B2 | 0.056 | 0.062 | 0.065 | 0.064 | 0.072 | 0.073 | 0.045 | **0.001** |  |  |  |
| B3 | 0.049 | 0.060 | 0.063 | 0.062 | 0.067 | 0.068 | 0.046 | 0.018 | **0.000** |  |  |
| B4 | 0.054 | 0.061 | 0.064 | 0.060 | 0.070 | 0.071 | 0.044 | 0.022 | 0.015 | **0.000** |  |
| B5 | 0.060 | 0.066 | 0.069 | 0.068 | 0.071 | 0.069 | 0.052 | 0.043 | 0.043 | 0.042 | **0.002** |
