# Supplementary figures and images for: Ecological niche and phylogeography elucidate complex biogeographic patterns in Loxosceles rufescens (Araneae, Sicariidae) in the Mediterranean Basin
Source: BMC Evol Biol. 2014 Oct 9;14:195. doi: 10.1186/s12862-014-0195-y (PMC4236462; doi:10.1186/s12862-014-0195-y)

Total diversity ( $H_T$ )Haplotype Richness ( $H_R$ )

Rarity Index (R)

All

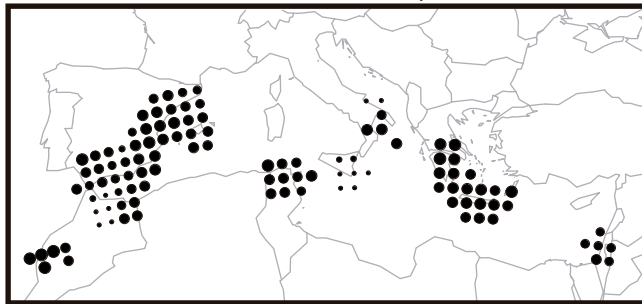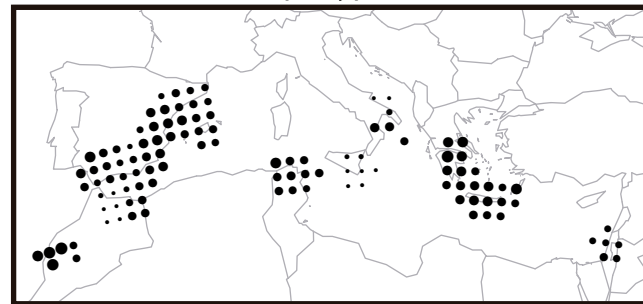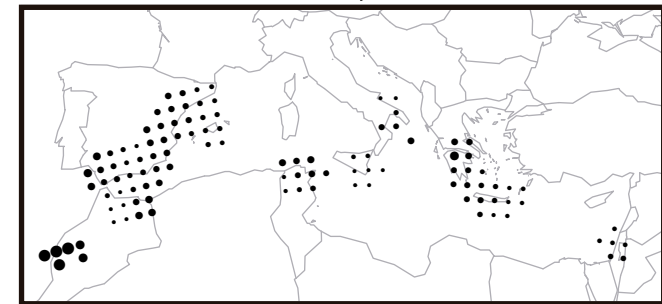

A6

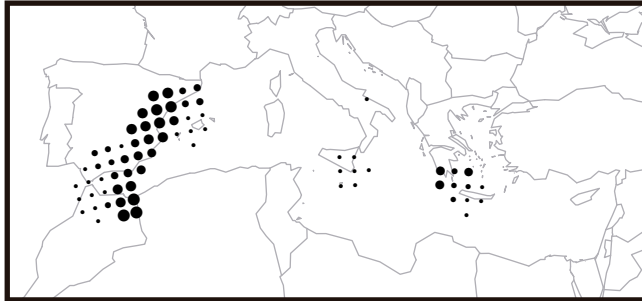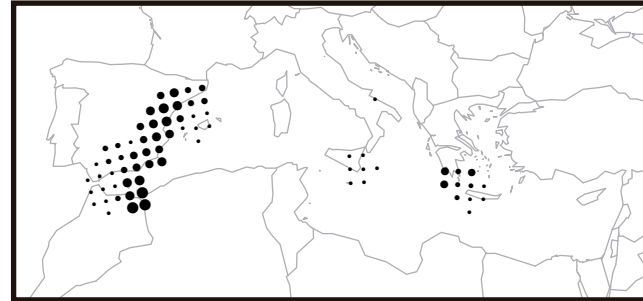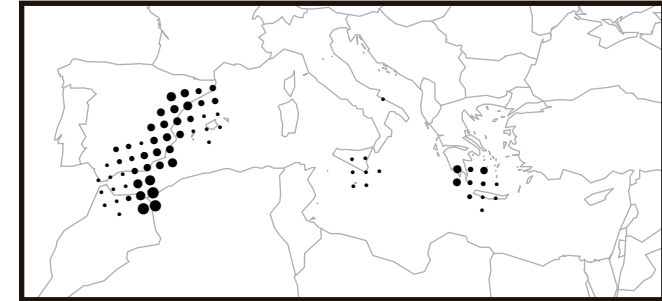

B2

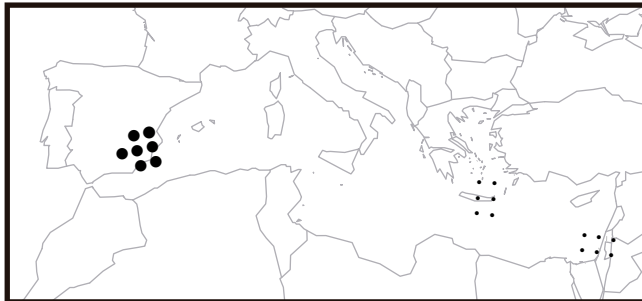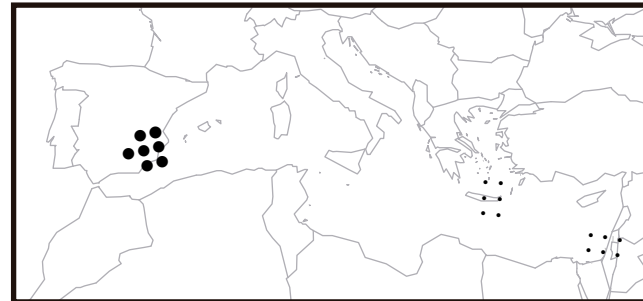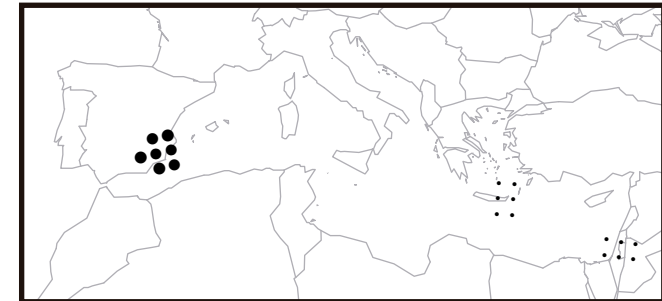

B4

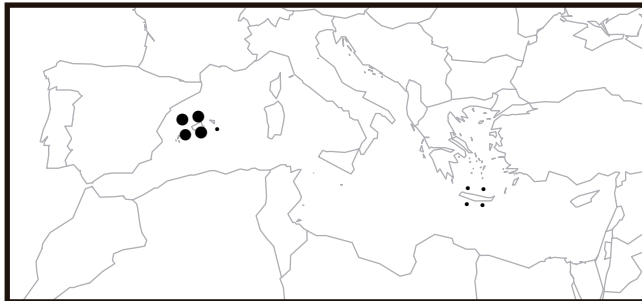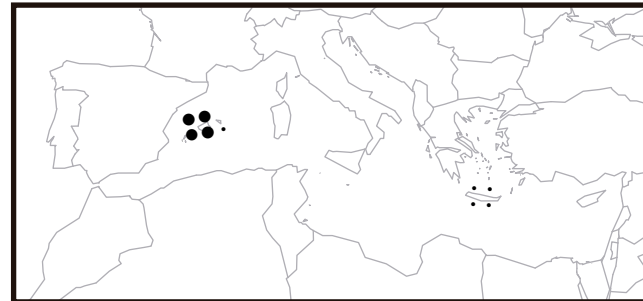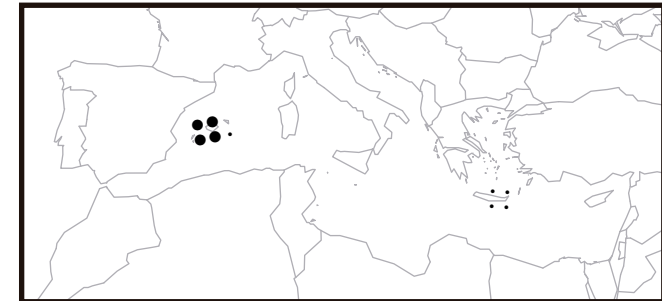

B5

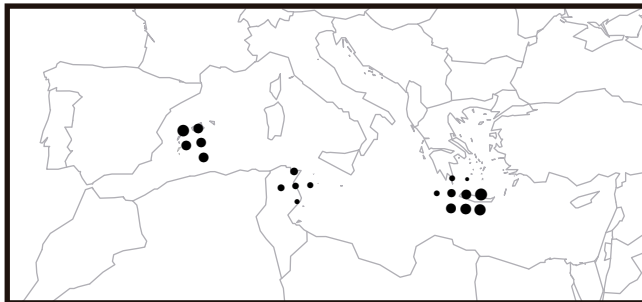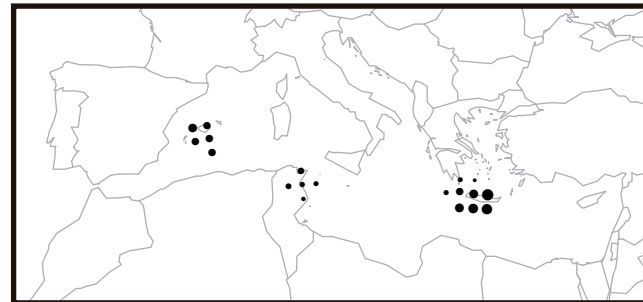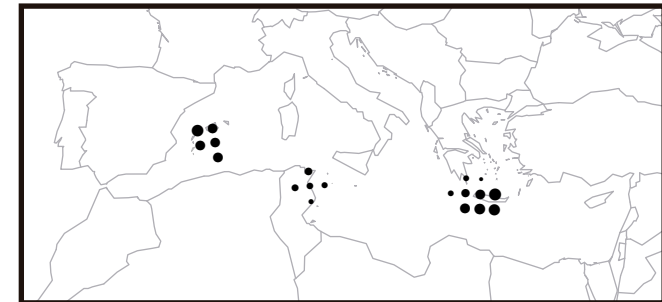

Supplement: Additional file 4: — Genetic diversity of Loxosceles rufescens in the Mediterranean. Total diversity (HT), haplotype richness (HR) and rarity index (R) are represented for L. rufescens (all) and separately for lineages A6, B2, B4 and B5. Diversity statistics are computed by considering samples located within a perimeter around a grid point. We set grid points every 100 km in latitude and longitude, and computations were conducted across random sets of 5 individuals, bootstrapping 1000 times. [file 12862_2014_195_MOESM4_ESM.pdf]

Present

mid-Holocene (~6 Ka)

Last Glacial Maximum (~21 Ka)

CCSM

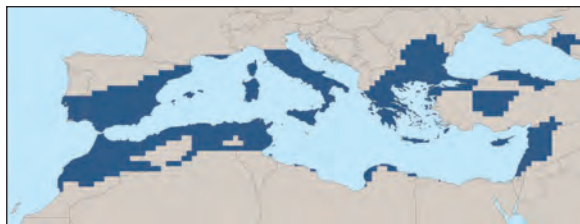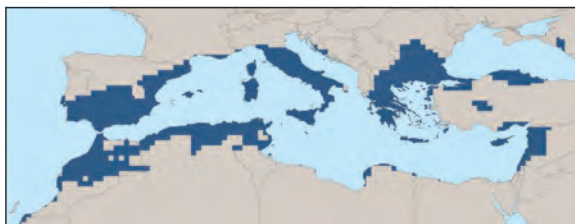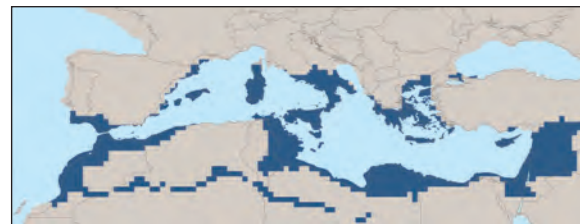

CNRM

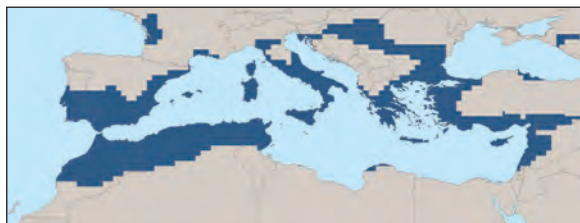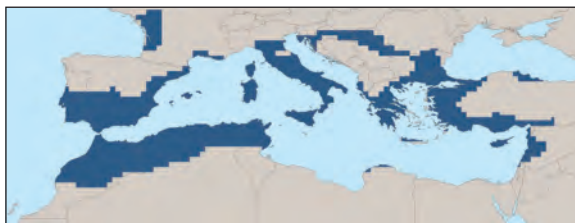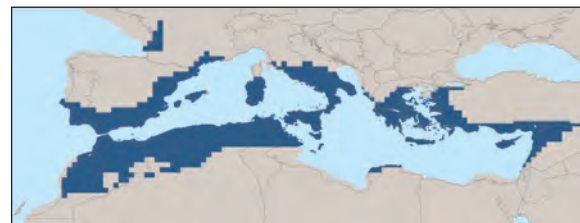

COSMOS

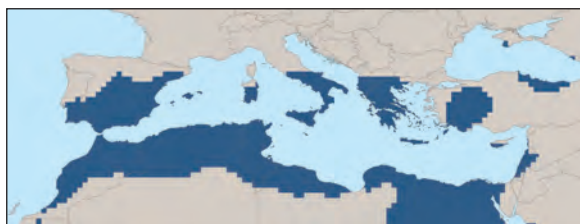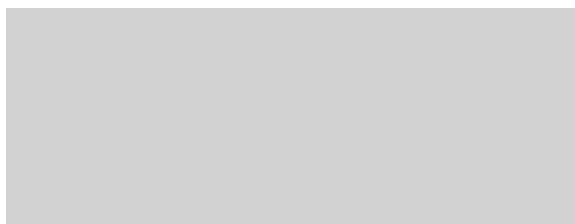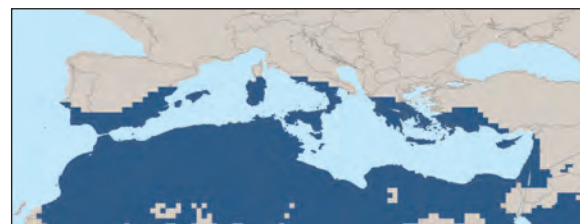

GISS

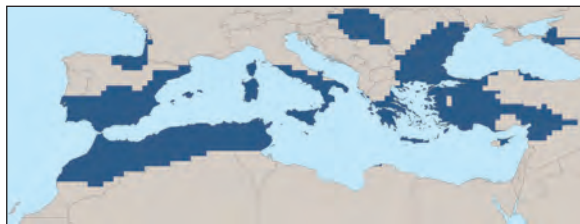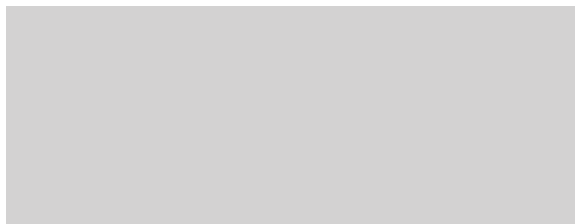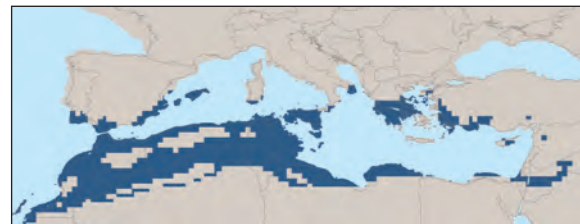

IPSL

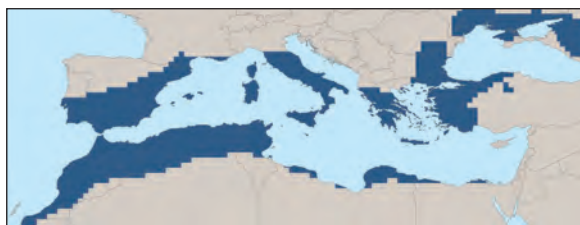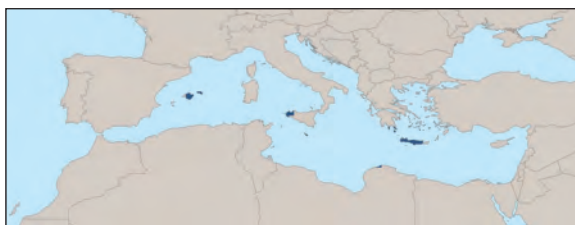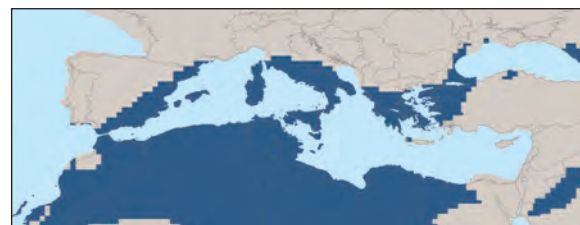

MIROC

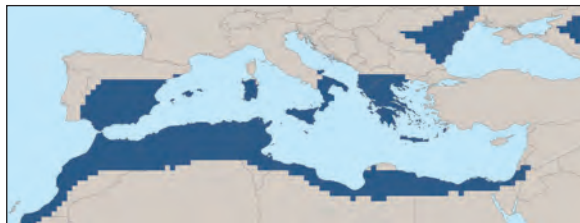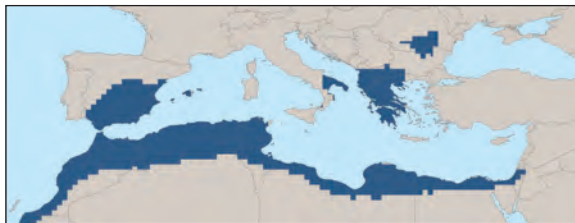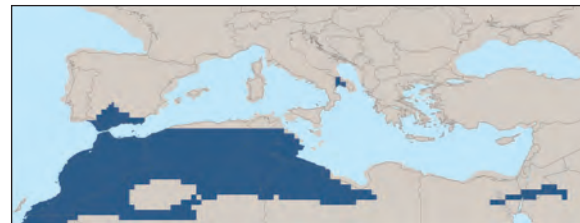

MPI

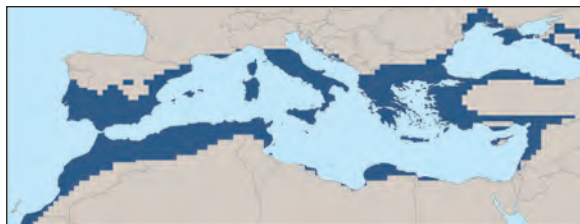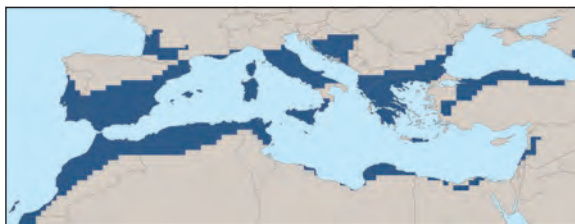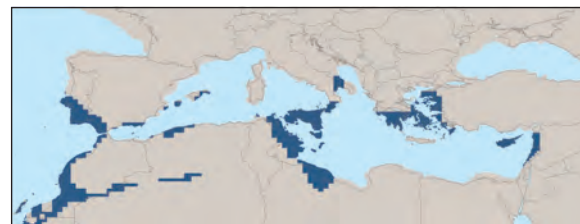

MRI

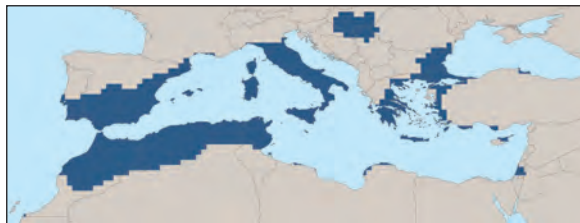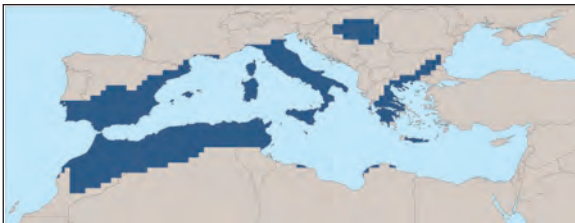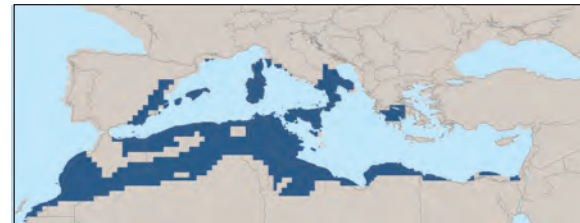

Supplement: Additional file 7: — Ecological niche modelling results for each atmosphere–ocean general circulation model (AOGCM). Ecological niche modelling results for each AOGCM in each time slice (Last Glacial Maximum, mid-Holocene, and present). [file 12862_2014_195_MOESM7_ESM.pdf]
