# Supplementary material for: Ecological niche and phylogeography elucidate complex biogeographic patterns in Loxosceles rufescens (Araneae, Sicariidae) in the Mediterranean Basin
Source: BMC Evol Biol. 2014 Oct 9;14:195. doi: 10.1186/s12862-014-0195-y (PMC4236462; doi:10.1186/s12862-014-0195-y)
Supplement: Additional file 5: — Dating analysis results. Dating analysis results in Ma obtained from two different cox1 rates: (1) using the rate for Parachtes, and (2) using a mean rate obtained for Loxosceles. [file 12862_2014_195_MOESM5_ESM.docx]

Additional file 5: Dating analysis results in Ma obtained from two different *cox1* especific rates; (1) using *Parachtes* rate and (2) using mean rate obtained for *Loxosceles*.

| mrca | 1 | 2 |
| --- | --- | --- |
| A-B | 1.968 (1.322-2.698) | 0.356 (0.243-0.487) |
| A | 1.606 (1.021-2.248) | 0.291 (0.192-0.413) |
| A6-A5-A4-A3-A2 | 0.936 (0.588-1.334) | 0.169 (0.107-0.238) |
| A6-A5-A4-A3 | 0.857 (0.547-1.218) | 0.155 (0.099-0.216) |
| A6-A5-A4 | 0.75 (0.47-1.074) | 0.136 (0.859-0.191) |
| A6-A5 | 0.25 (0.099-0.423) | 0.046 (0.184-0.078) |
| B | 1.2631 (0.76-1.885) | 0.228 (0.136-0.334) |
| B4-B3-B2-B1 | 1.096 (0.613-1.618) | 0.197 (0.113-0.29) |
| B4-B3-B2 | 0.499 (0.235-0.798) | 0.09 (0.045-0.145) |
| B4-B3 | 0.313 (0.125-0.537) | 0.057 (0.021-0.096) |
