# Supplementary material for: Ecological niche and phylogeography elucidate complex biogeographic patterns in Loxosceles rufescens (Araneae, Sicariidae) in the Mediterranean Basin
Source: BMC Evol Biol. 2014 Oct 9;14:195. doi: 10.1186/s12862-014-0195-y (PMC4236462; doi:10.1186/s12862-014-0195-y)
Supplement: Additional file 6: — Partial ROC analyses. Partial ROC tests (P < 0.05) using two data subsets with 1000 replicates. [file 12862_2014_195_MOESM6_ESM.doc]

Additional file 6: Partial ROC tests (*P* < 0.05) using two data subsets, with 1000 replicates.

| AOGCM | Data subset | 0.99 |
| --- | --- | --- |
| CCSM | Set 1 | 0.0047 |
|  | Set 2 | 0.0002 |
| CNRM | Set 1 | 0.0023 |
|  | Set 2 | 0.0002 |
| COSMOS | Set 1 | 0.0001 |
|  | Set 2 | 0.0080 |
| GISS | Set 1 | 0.0048 |
|  | Set 2 | 0.0032 |
| IPSL | Set 1 | 0.0000 |
|  | Set 2 | 0.0001 |
| MIROC | Set 1 | 0.0000 |
|  | Set 2 | 0.0001 |
| MPI | Set 1 | 0.1526 |
|  | Set 2 | 0.0211 |
| MRI | Set 1 | 0.0029 |
|  | Set 2 | 0.0073 |
